# Rock slope stability analysis of a limestone quarry in a case study of a National Cement Factory in Eastern Ethiopia

**DOI:** 10.1038/s41598-024-69196-8

**Published:** 2024-08-09

**Authors:** Getaneh Bezie, Endalu Tadele Chala, Nagessa Zerihun Jilo, Sisay Birhanu, Kalkidan Kefale Berta, Siraj Mulugeta Assefa, Biruk Gissila

**Affiliations:** 1 Department of Civil Engineering, Institute of Technology, Dire Dawa University, Dire Dawa, Ethiopia; 2 Department of Civil Engineering, College of Engineering, Addis Ababa Science and Technology University, Addis Ababa, Ethiopia; 3 Department of Civil Engineering, School of Civil and Environmental Engineering, Ambo University Woliso Campus, Woliso, Ethiopia; 4 O-Mining Group, Sengatera, Lideta Subcity, Addis Ababa, Ethiopia

**Keywords:** Geometric profile, Kinematic analysis, Numerical analysis, Parametric study, Quarry cut slope, Rock slope failure, Civil engineering, Natural hazards

## Abstract

Rock slope failures pose significant challenges in geotechnical engineering due to the intricate nature of rock masses, discontinuities, and various destabilizing factors during and after excavation. In mining industries, such as national cement factories, multi-benched excavation systems are commonly used for quarrying. However, cut slopes are often designed with steep angles to maximize economic benefits, inadvertently neglecting critical slope stability issues. This oversight can lead to slope instability, endangering human lives and property. This study focuses on analyzing the stability of existing quarry cut slopes, estimating their final depth, and conducting a parametric study of geometric profiles including bench height, width, face angle, and rump width. Kinematic analysis helps identify potential failure modes. The results reveal that the existing quarry cut slope is prone to toppling, wedge failure, and planar failure with probabilities of 42.68%, 19.53%, and 14.23%, respectively. Numerical modeling using the finite element method (Phase2 8.0 software) was performed under both static and dynamic loading conditions. The shear reduction factor (SRF) of the existing quarry cut slope was 1.01 under static loading and 0.86 under dynamic loading. Similarly, for the estimated depth, the SRF was 0.82 under static loading and 0.7 under dynamic loading. These values indicate that the slope stability falls significantly below the minimum acceptable SRF, rendering it unstable. The parametric study highlights the face angle of the bench as the most influential parameter in slope stability. By adjusting the bench face angle from 90° to 75°, 70°, and 65°, the SRF increased by 31.6%, 35.4%, and 37.9%, respectively. Among these, a 70° bench face angle is recommended for optimal stability with a SRF of 1.27 under static loading and 1.18 under dynamic loading.

## Introduction

Pit slope stability is crucial in mining and quarrying operations within geotechnical engineering projects, as slope failures can have severe consequences. According to Ali et al.^1^, rock slope failures in both man-made excavations and natural slopes include rock falls, overall slope instability, landslides, and failures in open-pit mines. Ifelola and Bassey^2^ state that slope failures mainly occur due to gravitational forces, geological and geo-mechanical properties, natural hazards, and technical issues during excavation. These failures can happen unexpectedly, either slowly or suddenly, leading to loss of life and property^3,4^.

Ifelola and Bassey^2^ generalize that unstable open-pit slope faces can lead to slope failures. The primary cause of these failures is the presence of unfavorably oriented discontinuities (joints, faults, bedding planes, foliation, and shear zones) that act as failure planes, causing plane, wedge, toppling, and circular failures^5–7^.

Slope stability analysis is essential for the success of engineering construction and significantly influences engineering investment and efficiency. Nowadays, these analyses are performed using advanced computer optimization techniques rather than hand calculations^8,9^. These methods are suitable for both regular slope stability problems and complex slope geometries, providing approximate results^10–12^. Monsalve et al.^13^ emphasize the importance of stability analysis for the safety of open-pit quarry operations.

Slope stability analyses are conducted to assess, compute, and design economically safe slopes, whether man-made or natural, under equilibrium conditions. Various methods are used for stability analysis, including the kinematic method, probabilistic method, limit equilibrium methods, finite element method (FEM), finite difference method (FDM), and discrete element method^14,15^. Kinematic slope analysis for open-pit mines evaluates the potential for translational failures by examining the geometry and orientation of discontinuities such as joints and faults^16^. This method uses stereographic projection techniques to identify critical failure planes and wedges^17^. It is particularly useful for assessing the stability of rock masses where discontinuities play a significant role^16^. Recent studies emphasize its importance in ensuring safe and efficient mine operations^18^.

Stability problems in open-pit mines often involve complexities that are not easily addressed by limit equilibrium methods. Fredj et al.^19^ and Singh et al.^20^ suggest using numerical methods, such as FEM and FDM, for more comprehensive and accurate slope stability analyses. The main advantage of numerical methods is that they do not require assumptions about the shape, direction, and location of slip surfaces^7,21^.

The study aimed to conduct a comprehensive slope stability analysis for the limestone quarry at the National Cement Factory in Eastern Ethiopia. This analysis was performed using Dips 6 for kinematic analysis and Phase2 for numerical modeling. The study also evaluated the excavatability of the limestone to optimize the quarrying process. By integrating the results of these analyses, the research provided recommendations for safe slope design, excavation methods, and potential reinforcement measures to enhance the stability and productivity of the limestone quarry.

## Description of the study area

**Figure 1:**
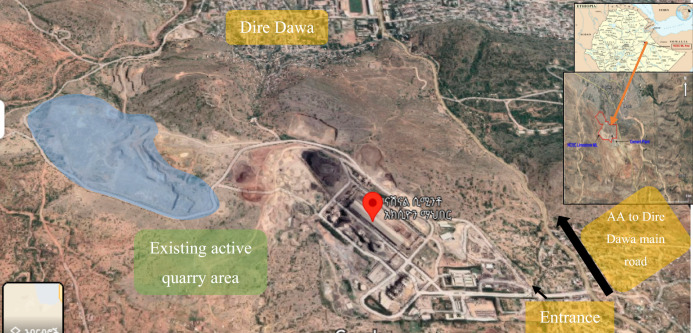
Location and accessibility of the study area (https://earth.google.com/web/search/national+cement+dire+dawa/@9.57220323,41.85211395,1303.69352672a,2499.40797394d,35y,0h,0t,0r/data=CigiJgokCQjtmrVONxdAEaO449W84BZAGZ7BPcn3e0NAIXqkhe2haENAOgMKATA).

The National Cement Share Company is located in the eastern part of Ethiopia, at the entrance of the city of Dire Dawa, 515 km from the capital of Addis Ababa. The area is bounded by geographical coordinates of latitude 9° 34′ 06.7′′ N to 9° 35′ 12.1′′ N and longitude 41° 50′ 21.8′′ E to 41° 51′ 35.0′′ E in eastern Ethiopia (Fig. 1).

### Field description of the study area

**Figure 2:**
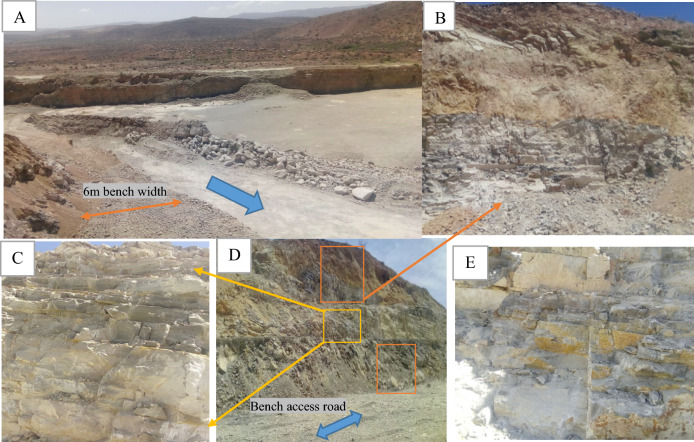
Typical quarries cut slope section of the open pit slope surface (**A**–**E**).

The National Cement Factory in Ethiopia, a major cement producer, operates a limestone quarry using open-pit mining with a multi-bench system. The extraction process involves drilling and blasting to remove the limestone, which is challenging. They conduct blasting once or twice weekly, yielding 4000–5000 tons daily for crushing. Each blast creates new quarry slopes, with the quarry reaching over 56 m in height, benches 6 m wide, and 12 m high. There also a 10 m wide access road for transport and safety. Signs of slope instability, like tension cracks and rock falls, have been observed (Fig. 2). The goal is to reach a depth of 150 m.

In open-pit mining, the drilling and blasting process is as follows:

1. Drilling: Holes are drilled with a diameter of 125 mm. The depth (load) is 3.5 m, and the spacing between holes ranges from 1.0 to 2.0 times the depth.
2. Blasting: Uses millisecond delay and nonelectric detonators with a mix of ANFO and emulsion/slurry explosives at an 80:20 ratio.

#### Geology and geomaterial of the study area

**Figure 3:**
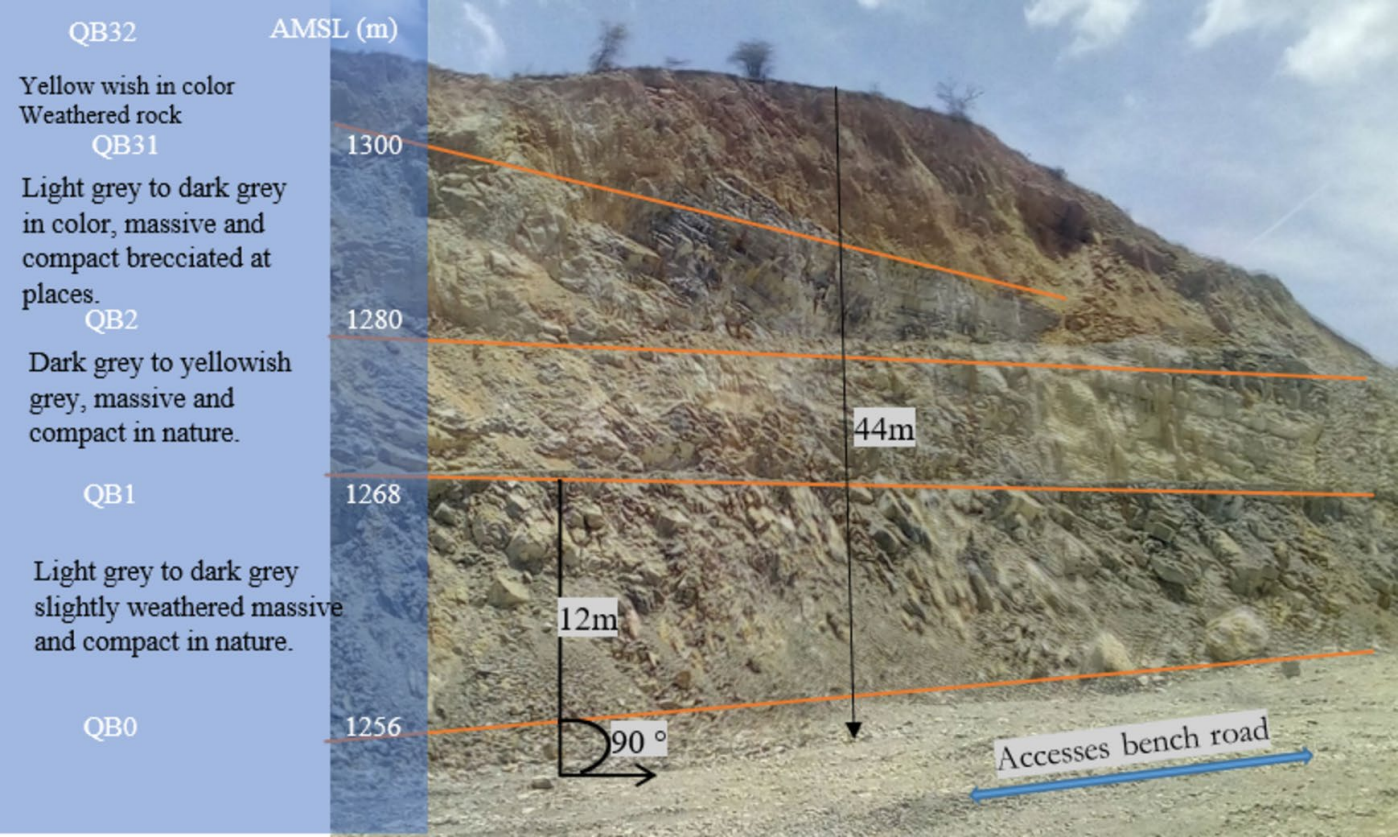
Geological and geomaterial future of slope cut sections.

The Awash River Basin in Ethiopia is geologically diverse, with rock formations ranging from the Precambrian to the Quaternary period. Notable formations include the Hamanlei Formation, characterized by gray-white and oolitic limestone with shale layers, and the Amba Arandam Formation, known for its fossil-rich gray limestone and upper layers of dolomite (Fig. 3). These formations reflect the basin history of volcanic activity and tectonic changes.

The study area is characterized by massive, fine-grained limestone that ranges in color from light to dark gray, occasionally displaying yellow or black tones. The presence of color variations does not influence its chemical properties. While the limestone exhibits surface weathering features like hollows, it lacks karstic formations. It is also associated with Upper Cretaceous sandstone and intersected by basalt and rhyolite formations, which are fine-grained and show signs of weathering.

#### Seismic effects on the study area

Seismic waves from earthquakes pose risks to structures by potentially widening structural discontinuities, impacting rock mass strength^15^. Ethiopia is categorized into five seismic zones based on local hazards, following the European Norm^22^. Table 1 lists the maximum ground acceleration for each zone.

**Table 1:** Bedrock acceleration ratio α_o_^22^.

Zones	0	1	2	3	4	5	
α_o_ = a_g_/g	ES EN 1998–1,2015	0	0.04	0.07	0.1	0.15	0.2

Located near seismic hotspots, the study area falls within Zone 3, a medium-risk category with a maximum bedrock acceleration coefficient of 0.1. It encompasses the Wonji and Adama faults, historical earthquake sources with PGA values from 0.1 to 0.115 g. Table 2 summarizes the seismic hazard zonation and PGA for the area.

**Table 2:** Summary of the PGA of the bedrock in the study area.

City	Longitude	Latitude	PGA Value	
			Tabular listing^22^	Map
Dire Dawa	41.8389	9.5034	0.1	0.1–0.115

#### Effect of blasts on the study area

The stability of a rock mass is affected by its multiple joint structures and by earthquakes and explosion operations. Exploded explosives have two main effects in the field: they damage rocks or their compressive strength parameters and cause the slope to collapse when the shock wave propagates through the rock mass^23–26^. Different researchers have derived empirical equations for the peak particle velocity. One of the most widely used equations is the general formula proposed by Adhikari et al.^27^, which is given by Eq. (1).

1 $${\text{PPV = K}}\left( {\frac{{\text{D}}}{{\sqrt {\text{Q}} }}} \right)^{{\text{b}}}$$

where peak particle velocity (PPV) in millimeters per second is a function of the distance (D) in m between the sensor and the blast, the maximum charge per delay (Q) in kg, and the site constants (K and b). This relationship is known as the scaled distance. Using blast vibration data from various limestone quarries, each with different site constants and correlation coefficients, Adhikari et al.^27^ derived a general formula (Eq. 2).

2 $${\text{PPV = 137}}{.67 }\left( {{\text{D/}}\sqrt {\text{Q}} } \right)^{{ - {1}{\text{.188 }}}}$$

The vibration acceleration of a particle, expressed in units of gravitational acceleration (g), was computed using Eq. (3). This method, suggested by Djordjevic et al.^28^ and Salunkhe et al.^29^, is defined as:

3 $${\text{PPA (g) = 9}}{.115 }\left( {{\text{D/}}\sqrt {\text{Q}} } \right)^{{ - {1}{\text{.747}}}}$$

Vibration acceleration is determined using site constants, detailed in Eqs. (2) and (3). Blasting involves using millisecond delay and nonelectric detonators to simultaneously blast holes in 2 to 3 rows. Blasts occur once or twice weekly, using a mix of ANFO and emulsion/suspension explosives at an 80:20 ratio. The specific site parameters for blasting are listed in Table 3.

**Table 3:** Summary of blasting parameters for the study area.

Parameter	Unit	Values
Bulk density (Limestone) (p)	t/m^3^	2.50
Blasting agent density (ANFO: emulsion)	(g/cc) 80: 20	0.88 and 1.2
Hole Depth (H)	(m)	12.0
Hole Diameter (d)	(mm)	115
Burden (B)	(m)	3.50
Spacing (S)	(m)	5.50
Stemming (T)	(m)	3.00
Powder Column	(PC)	9.00
Loading Density	(LD)	9.14
Charge Weight	(CW)/hole	82.26
Powder Factor	(PF)	7.02

Table 3 provides a comprehensive summary of the blasting parameters for the study area, which is likely related to quarry or mining operations. The bulk density of limestone is 2.50 t/m^3^, while the blasting agent (ANFO: Emulsion) has densities of 0.88 and 1.2 g/cc. The hole specifications include a depth of 12.0 m and a diameter of 115 mm. The blasting geometry parameters are a burden of 3.50 m, spacing of 3.00 m, and stemming length of 5.00 m. Additionally, the powder column length is 9.00 m, and the load charge weight per hole is (82.26 CW/hole).

Therefore, there is an explosion charge of 82 kg per hole and a minimum distance of 65 m between the sensor and the blast. Therefore, the maximum particle velocity (PPV) was 18.08 m/s, and a vibration acceleration PPA_(g)_ of 0.299 g was used as the input PGA related to blast excavation. These parameters are crucial for planning and executing effective blasting operations.

## Materials and methods

The study relied on primary data collected during a field investigation of a limestone quarry cut slope. This data included quarry geometry, rock mass discontinuity details, and dip/dip direction orientations. The investigation involved Schmidt rebound hammer tests and analyses of geological structures, discontinuity orientation, rock mass strength, and slope geometry^30,31^.

The secondary data for this study included geological maps, earthquake effects, and rock strength parameters. These data were derived from the literature and empirical correlations. The geological strength index (GSI) was used to determine the rock mass and the deformation modulus. The Ethiopian building codes standard provided the coefficient of horizontal acceleration due to the earthquake for the study area^22,32^. Additionally, topographic maps, geological exploration data, and various investigations were obtained from the National Center for Seismology and Crustal Studies, as previously reported by Holtec^33^.

**Figure 4:**
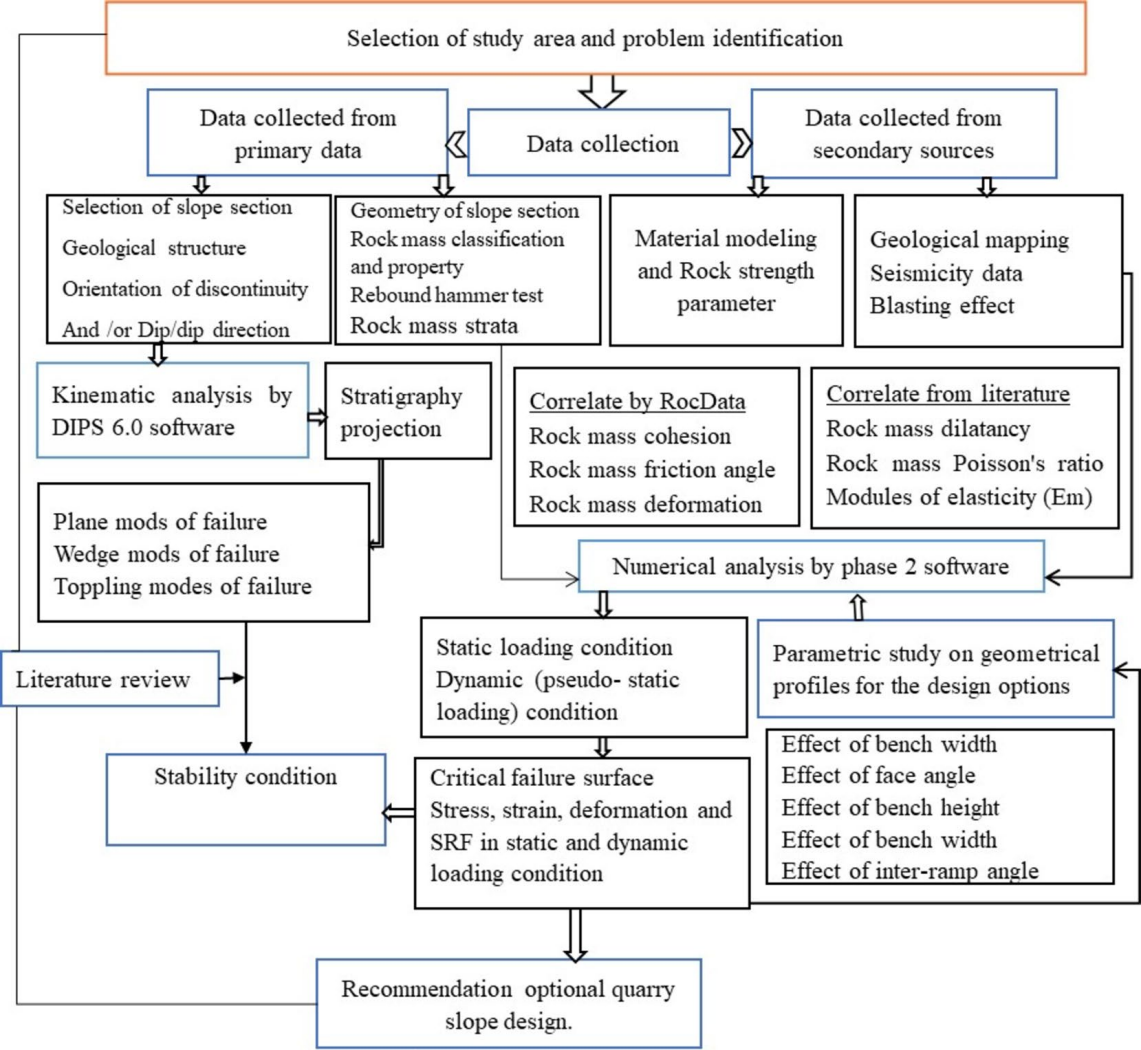
Technical scheme of the block diagram research methodology.

Stability analysis, encompassing both kinematic and numerical modeling, utilized diverse data sources. Kinematic analysis, following Rocscience^34^, identified potential failure modes in geometric profiles. Numerical modeling assessed the critical quarry cut slope, corroborated by case studies. A parametric study examined the influence of geometry on slope stability, leading to an optimal design prioritizing safety and resource utilization. The methodology is summarized in Fig. 4.

### Data collection

Slope stability analysis, a multifaceted geotechnical challenge, hinges on numerous verified factors^35,36^. This study incorporated primary data from fieldwork and tests, supplemented by secondary data from literature and field-based correlations. Field measurements informed the kinematic analysis, while numerical modeling relied on comprehensive field data.

#### Field investigation

**Figure 5:**
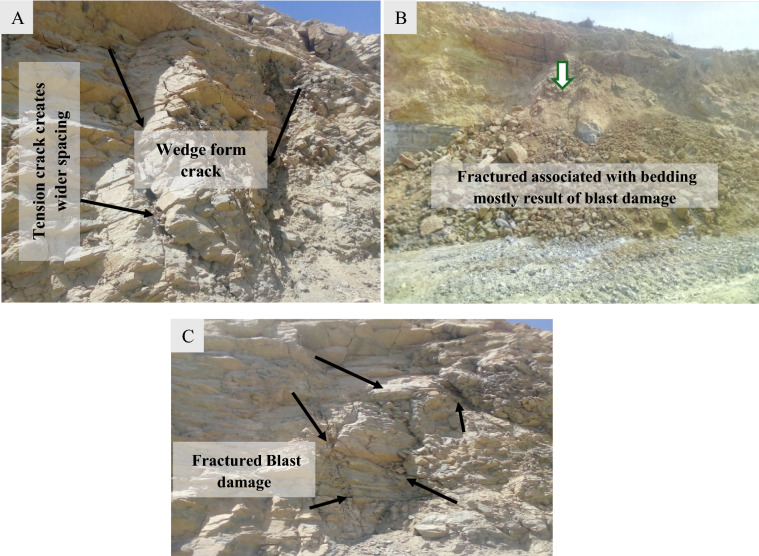
Failure manifestations: (**A**) tension cracks, (**B**) sliding failure, and (**C**) fractures on the section of the cut slope of the quarry.

The field study analyzed geological structures and rock mass properties to evaluate potential slope failures and compute shear strength. Failures were linked to weathering and vibrations, as shown in Figs. 5A,B, with additional fractures observed in Fig. 5C. The study also noted diverse orientations of discontinuities, changes in rock mass appearance, and weathering-related strength reduction.

Investigations of the quarry slope revealed varying degrees of weathering, with the most pronounced effects on the upper layers, indicating potential instability. The geological orientation of discontinuities, informed by Ifelola and Bassey^2^ and Saadoun et al.^25^, was systematically measured across the slope using a Brunton compass. The steep dips observed suggest a significant impact on slope stability.

#### Characteristics of the rock mass during field inspection

**Figure 6:**
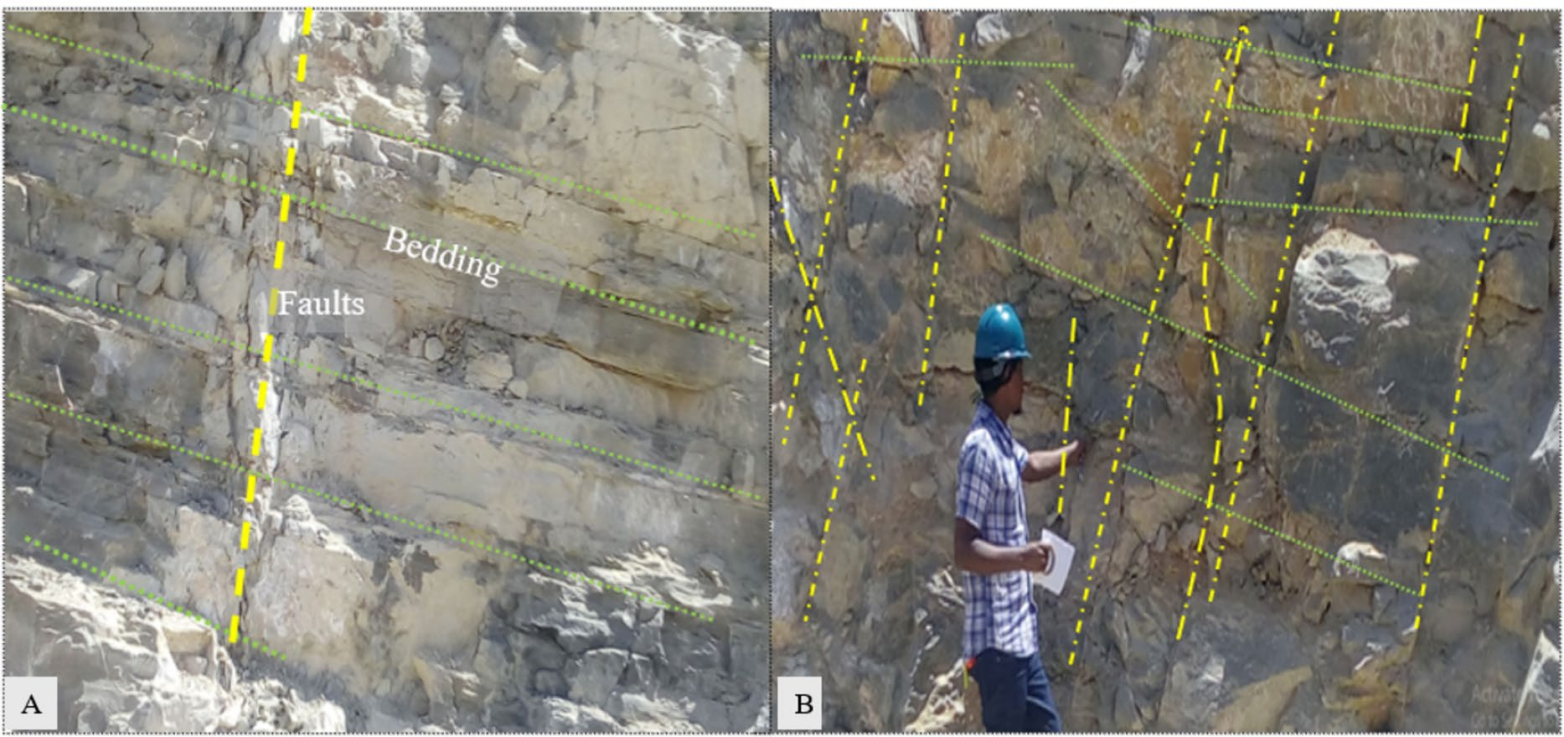
Estimation of rock mass characteristics by field inspection by authors (**A**, **B**).

The study focused on assessing the rock mass properties by examining its structure and characteristics. Notably disintegrated and blocky, the rock mass featured multiple discontinuities, depicted in Fig. 6A. Figure 6B illustrates how these discontinuities transitioned the rock from intact to blocky. Field data facilitated the estimation of key parameters, including the geological strength index (GSI), the intact rock coefficient (mi), and the disturbance factor (D).

The geological strength index (GSI) was estimated based on the geological structure, including surface conditions and rock block interlocking, following references Marinos et al.^37^ and Wyllie^38^. The rock mass exhibited a blocky and disintegrated structure with varying degrees of interlocking. Surface conditions ranged from poor to very good. The disturbance factor (D) accounted for excavation impacts; while most slopes underwent controlled blasting, the top layer experienced uncontrolled rock mass damage. The intact rock coefficient (mi) was determined from the rock physical properties, primarily sedimentary limestone with textures ranging from fine to coarse. Refer to Fig. 6 for a visual summary of these rock mass properties.

#### Field test and orientation measurement

Kinematic analysis utilized field data on dip, dip direction, and strike of geological features such as joints and discontinuities. Measurements were taken with a geological compass and analyzed alongside slope dips and friction cone angles. Weathering impact on rock strength was considered with field tests determining site-specific strength^39,40^. The uniaxial compressive strength (σ_ci_) was estimated using the Schmidt hammer test.

#### Discontinuity orientation dips/dip direction

The study utilized joint and bedding orientation data from^19,41^. The scanline method, as described by Ifelola and Bassey^2^, was used to measure rock mass discontinuities caused by excavation. In the study area, limestone bedding varies from 100° to 400° toward the south-southeast^33^. Field measurements confirmed a minimum dip value of 12° and a dip direction of 102°.

Discontinuity measurements from the quarry cut face were conducted in accordance with^19,26^. A line of 50 m was established perpendicular to the cut face to plot intersecting discontinuities. Using a geological compass, the maximum dip angle and direction were recorded. Data on discontinuities were collected from over 400 rock masses at the active bench face and its lithology. The field investigation, adhering to international standards, included hammer tests on five selected quarry cut slopes^30,42^.

The rebound number from Schmidt hammer tests was used to estimate the uniaxial compressive strength (UCS) by correlating it with the rock mass dry density^43^. Additionally, empirical correlations allowed for UCS estimation (σci) through a logarithmic equation based on the rock unit weight and average rebound number, as detailed in Eq. (4)^42^.

4 $${\text{log }}\left( {{\upsigma }_{{{\text{ci}}}} } \right) = { 0}{\text{.00088}}*{\upgamma }*{\text{R + 1}}{.01}$$

where (σ_ci_) is in MPa, (γdry) is the dry density of the rock in kN/m^3^, and (R) is the average schmidt hammer value. Therefore, uniaxial compressive strength (σci) values in Table 4, the maximum value was obtained based on Barton^42^ and used as the input for analysis purposes. Table 4 summarizes the uniaxial compressive strength (σ_ci_) results.

**Table 4:** Summary of the uniaxial completion strength of the rock mass.

Slope section	Average rebound number (R)	Dry density (kN/m^3^)	(σ_ci_), Barton^42^ (MPa)
QB32	23.5	24.5	32.4
QB31	45.2	24.5	97.3
QB2	50.2	24.5	123.8
QB1	53.7	24.5	147.1
QB0	54.3	24.5	151.9

#### Shear strength property of the rock mass

The shear strength of the rock mass, determined from its characterization, is computed as per RocData^44^. Figure 3 displays a sample analysis for the quarry cut slope (QB32), while Table 5 presents the summarized shear strength data for all quarry layers. GSI from field observations, which considers the degree of weathering and fracturing, UCS derived from point load tests, mi based on the site lithology, and D as the disturbance factor. By incorporating these parameters into RocData, we can accurately determine the Mohr–Coulomb strength parameters for the rock mass.

**Table 5:** Summary of the representation of the rock mass shear strength parameters.

Slope section	UCS (MPa)	Hock‒Brown classification			Hoek‒Brown criterion			Mohr–Coulomb criterion		Rock mass parameters (MPa)		
		GSI	M_i_	D	M_b_	s	a	C (MPa)	Φ (°)	Sigt	Sigc	Em
QB32	32.9	20	8	1	0.026	1.6e−06	0.544	0.060	13.23	− 0.002	0.023	510.00
QB31	87.4	60	10	0.7	1.110	0.003	0.503	0.849	49.88	− 0.239	4.737	10,806.1
QB2	123.8	78	10	0.7	2.986	0.041	0.501	3.82	54.62	− 1.710	25.08	32,577.2
QB1	147.1	80	12	0.7	3.999	0.055	0.501	4.916	57.21	− 2.038	34.66	36,552.2
QB0	151.9	80	12	0.7	3.999	0.055	0.501	5.044	57.28	− 2.093	35.596	36,552.2

The Hoek–Brown criterion was used to calculate the rock deformation modulus, which in turn provided an estimate of its uniaxial compressive strength, as supported by empirical data^31^. The RocData^44^ software provided Eqs. (5a) and (5b), which yielded comparable results.

5a $${\text{For}}\, \, \sigma_{ci} \le { 1}00\,{\text{ MPa}}, \,{\text{E}}_{{\text{m}}} \left( {{\text{GPa}}} \right){ = }\left( {{1} - \frac{{\text{D}}}{{2}}} \right)\sqrt {\frac{{{\sigma }\,{\text{ci }}}}{{{100}}}} \cdot {10}^{{\left( {\frac{{\text{GSI - 10}}}{{{40}}}} \right)}}$$

and

5b $$\sigma_{ci} > { 1}00 \, \,{\text{MPa}}, \, \,{\text{Em}}\left( {{\text{GPa}}} \right){ = }\left( {{1} - \frac{{\text{D}}}{{2}}} \right) \cdot {10}^{{\left( {\frac{{\text{GSI - 10}}}{{{40}}}} \right)}}$$

Poisson’s ratio, a key mechanical parameter for rocks, is derived using the Hoek–Brown constant and the GSI, following the method outlined in^45^. This calculation is detailed in Table 6 and is used in Eq. (6) for analysis.

6 $${\upnu } = - {0}{\text{.002}}\,{\text{GSI}} - { 0}{\text{.003}}\,{\text{mi }} + { 0}{\text{.45}}$$

**Table 6:** Summary of module deformation and Poisson’s ratio Vasarhelyi^45^.

Layers slope section	UCS (MPa)	GSI and mi		D	E_m_ (GPa)	V
		GSI	mi			
QB32	32.9	20	8	1	0.5	0.393
QB31	87.4	60	10	0.7	10.8	0.307
QB2	123.8	78	10	0.7	32.6	0.271
QB1	147.1	80	12	0.7	36.6	0.261
QB0	151.9	80	12	0.7	36.6	0.261

### Modeling approaches and analysis

Analyzing rock slope stability is essential for creating secure excavations like quarries and road cuts, and for assessing natural slope balance. Experts concur that understanding failure modes via kinematic analysis and calculating safety factors with numerical analysis are crucial for stable quarry designs. This section examines these two key methods of slope stability analysis.

#### Modeling for kinematic analysis

**Figure 7:**
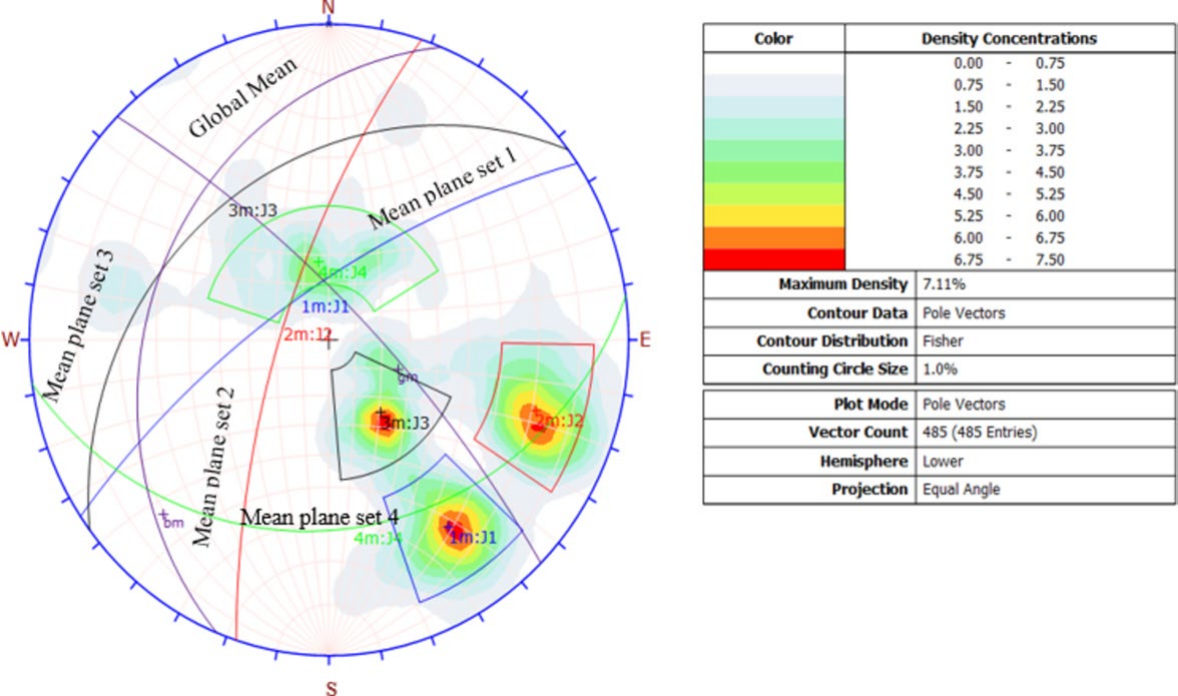
Stereographic projection of structural geology.

The study analyzed failure modes by assessing discontinuities and slope angles relative to friction limits. Using stereographic projection to translate 3D geological data into a 2D format, we visualized critical planes and lines^41^. Figure 7 illustrates this projection, mapping discontinuity orientations from field data in the target area. The accompanying Table 7 encapsulates these findings. Dip software^34^ was applied to evaluate key geological features and identify potential failure mechanisms within the lower hemisphere projection.

**Table 7:** Summary of the stratigraphic representation.

Joint set	Dip angle	Dip direction	
J1	19	146	Friction cone 54°
J2	18	108	Lateral limit 20%
J3	58	143	
J4	62	352	Slope D/DD 80/330

### Numerical modeling

In geotechnical engineering, numerical modeling, particularly the finite element method, is crucial for evaluating the stability of limestone quarry slopes, ensuring safety and economic viability^46,47^. This study used a discrete continuum model to simulate the behavior of jointed rock masses and employed Phase2 v8.0^48^ for finite element analysis of quarry slope profiles. The analysis assessed stability, calculated deformations, and determined safety factors under various loading conditions. The findings were corroborated by literature case studies, enhancing understanding of structural failure, deformation, and safety mechanisms.

#### Modeling with Phase2 8.0

The study applied the finite element method, transforming continuous problems into discrete ones for solution via matrix notation, as noted by Rao^49^. Gaussian elimination simplified the equation system into a triangular format for ease of calculation. Using Phase2, the study modeled four quarry cut slopes for FEM analysis, considering multistage excavation and blasting. The model excavation stage was predetermined, incorporating boundaries, geological conditions, and material properties. To enhance result accuracy, mesh density was increased, with mesh resolution being vital for FEM analysis accuracy. A plane strain model represented the slope geometry, and safety factors were calculated using shear strength reduction factor methods.

#### Geometric modeling

**Figure 8:**
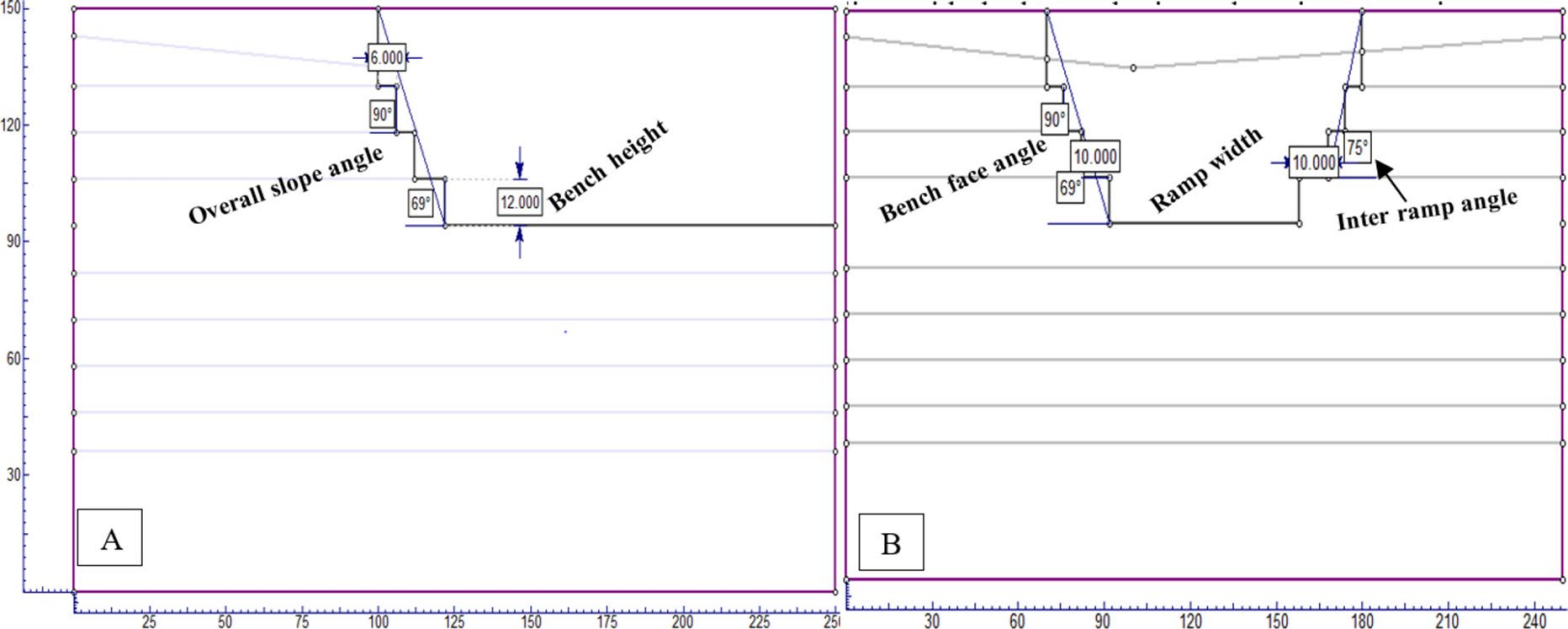
Geometrical profile of the existing quarry cut slope section (**A**) and open-pit quarry slope model (**B**).

The study utilized extensive geological cross-sections for modeling, with the largest horizontal section measuring about 254 m. It analyzed two scenarios with identical material boundaries, depicted in figures showing different quarry slope sections. The limestone, being dense and hard, necessitates blasting for extraction. Drilling is done vertically to a depth of 12 m, forming benches that stand nearly vertical to the ground. The models use a bench design with 12 m height, 6 m width, and a 90° bench face angle. The internal ramp has a 73° slope, and 10 m wide temporary roads connect the benches to the main highway, with the quarry overall slope at 67° degrees (Fig. 8).

**Figure 9:**
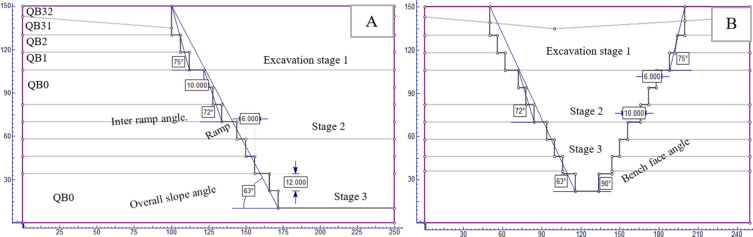
Geometrical profile for the ultimate depth of the quarry cut slope section (**A**) and open-pit quarry cut slope model (**B**.)

The NCSC limestone quarry is expanding, with each blast shaping new benches and elevating the slope. The goal is to reach a final depth of 150 m, optimizing mineral use while considering geological and stability factors. Excavation will proceed to a minimum width of 25 m at the base. The design includes benches 12 m high and 6 m wide, with an internal ramp angle between 72° and 73°, and an overall slope of 62° (Fig. 9). The quarry features three access roads for connectivity, and all models adhere to consistent parameters for structural integrity.

#### Geomechanical input properties

The outcome of the numerical analysis is influenced by the input parameters. For accurate simulation of block behavior, the Mohr–Coulomb model is employed, which requires precise mechanical properties of the slope material. These properties include cohesion (c), friction angle (ϕ), elastic modulus (E), Poisson’s ratio (ν), and unit weight (γ) for each layer of the quarry bench, as detailed in section “Modeling approaches and analysis”, field tests, and RocData software^44^.

The study applied the Mohr–Coulomb model to simulate the behavior of rock layers, treating them as ideal elasto-plastic materials. This means the rocks exhibit both elastic and plastic characteristics, with constant peak and residual strength parameters. Dilation, or the volume change during shearing, was considered, setting the angle of dilatancy, (ψ) = φ/8 for average quality rocks and zero for poor quality ones like the QB32 quarry bench. The effective rock parameters referenced in Table 8 from an earlier section were used for the modeling.

**Table 8:** Summary of the input data used in the quarry cut slope stability analysis.

Input material parameter	Unit	Quarry slope section				
		QB0	QB1	QB2	QB31	QB32
Unit weight (γ )	kN/m^3^	24.5	24.5	24.5	24.5	24.5
Cohesion (*c*)	MPa	5.04	4.92	3.82	0.849	0.06
Friction angle (ϕ)	Degree	57.21	57.28	54.62	49.88	13.23
Dilatancy (ψ)	Degree	7.2	7.2	6.8	6.2	0
Uniaxial compression strength (σ_ci_)	MPa	151.9	146.7	123.8	87.4	32.9
Tensile strength (σ_t_)	MPa	2.093	2.038	1.710	0.239	0.002
Young’s modulus (*E*_*m*_)	MPa	36,775.2	36,775.2	32,552.2	10,806.1	510
Poisson’s ratio (ν)	–	0.261	0.261	0.271	0.307	0.391
Hoek‒Brown constant (M_b)_	–	3.999	3.999	2.986	1.110	0.026
Hoek‒Brown constant (s)	–	0.055	0.055	0.041	0.003	1.6e−06
Hoek‒Brown constant (a)	–	0.501	0.501	0.501	0.503	0.544

#### Boundary conditions and discretization and mesh of the model

In the finite element model, we simulated real-world conditions by setting fixed boundaries to prevent rotation and movement along the x and y axes (Ux = Uy = 0), allowing vertical movement only. This setup ensured that during static analysis, the rock mass was vertically stable with negligible horizontal displacement.

**Figure 10:**
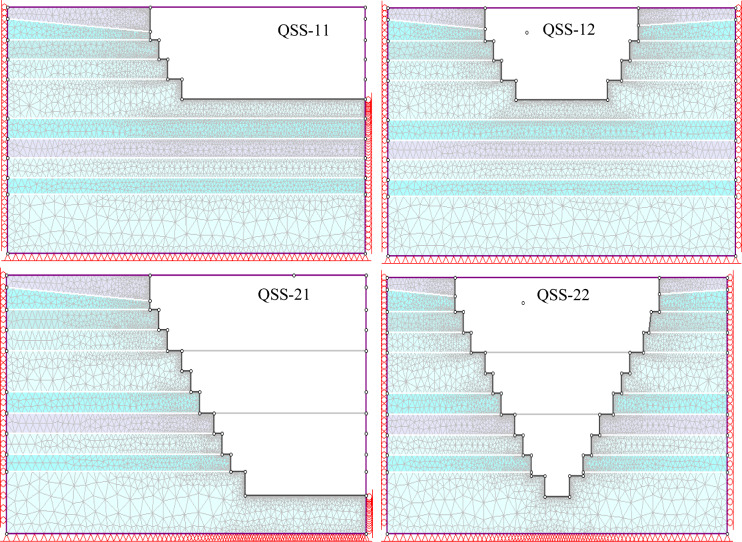
Mesh generation/discretization of the model into the finite element.

**Figure 11:**
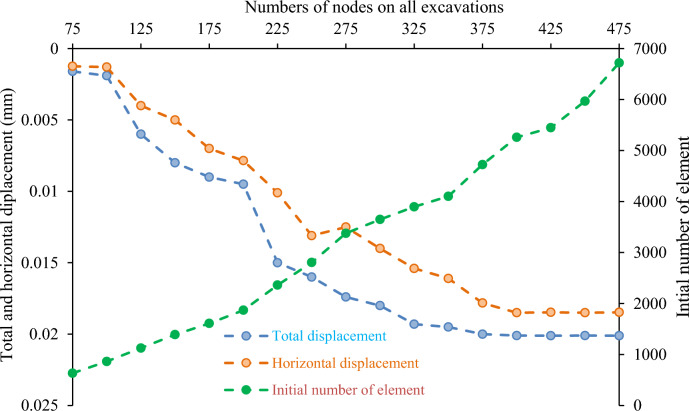
Sensitivity analysis of the deformation of the geometrical mode.

Finite element analysis breaks down a model into smaller interconnected units, or elements, to simulate the model behavior. This study utilized a 3-node triangular element with a gradation factor of 0.1 for detailed analysis (Fig. 10). The element shape and size influence the accuracy and computational time of the solution, with smaller elements yielding more precise results but requiring longer computation (Fig. 11).

## Results and discussion

The analysis aimed to assess the stability of rock slopes, explore potential failure types, gauge sensitivity to various triggers, and consider safe, reliable, and cost-effective design options. Additionally, it involved examining the geometry of a critical quarry slope to identify potential failure modes using kinematic analysis and stereographic projection, as well as evaluating the slope under static and dynamic conditions. It also included a discussion on choosing the best quarry slope design for safety and optimal resource use.

### Results of the kinematic analysis

The analysis identified potential failure modes, such as wedge, planar, and toppling failures, which are highly dependent on the quarry slope orientation. These modes, while not actual failures, indicate varying instability levels in certain quarry sections. The kinematic analysis indicated a 42.68% chance of toppling failure, with wedge and planar sliding failures at 19.53% and 14.23% likelihoods, respectively, demonstrating the varying stability of potential failure modes.

#### Wedge failure results

**Figure 12:**
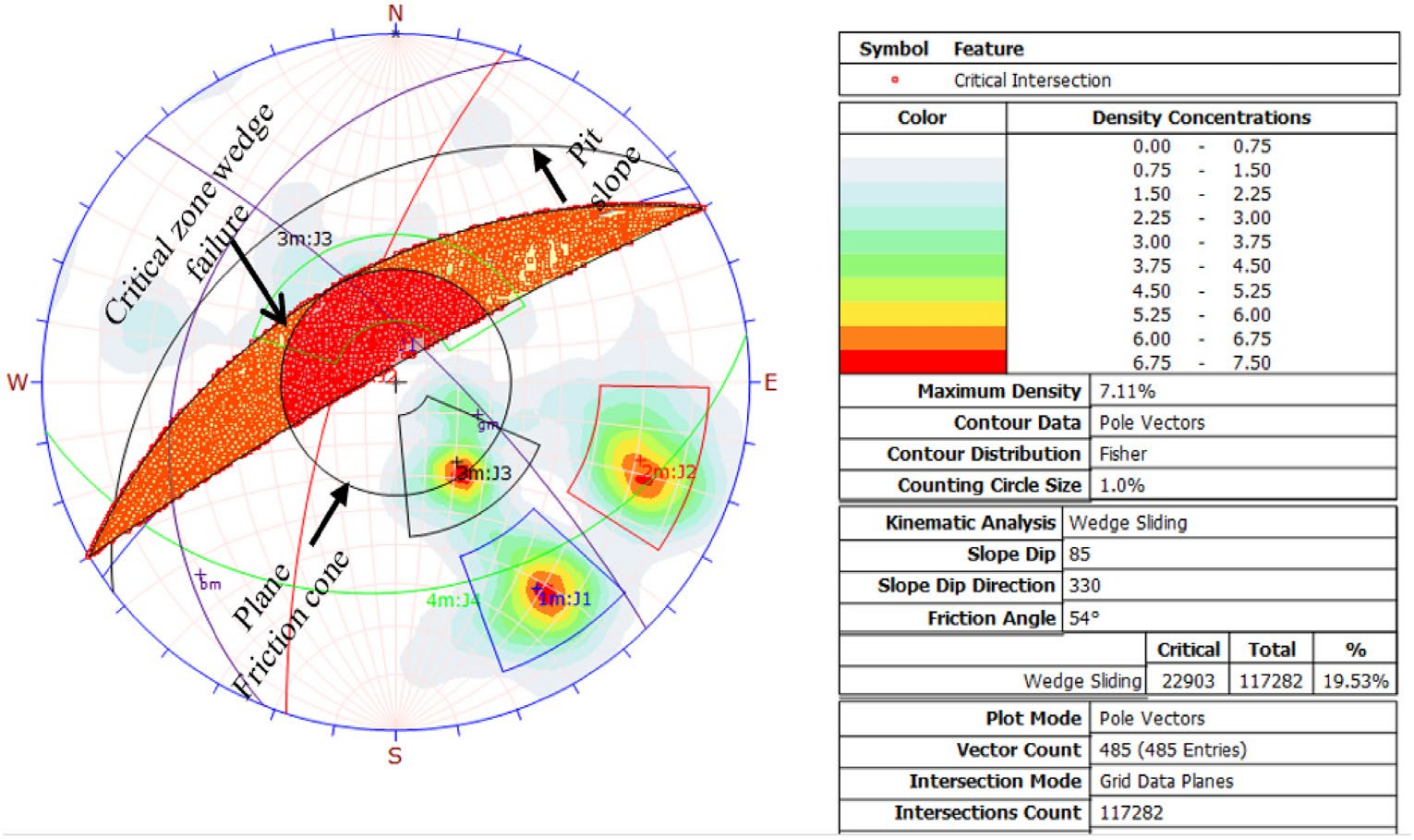
Wedge modes of failure from kinematic analysis.

Kinematic analysis suggests wedge failures are likely at discontinuity intersections, with 19.5% of over 117,282 checked points critical, as depicted in Fig. 12. Table 10 details that 53.4% of J1 and J2 joint sets, and 39.6% of J1 and J3, are in the wedge failure zone.

#### Planar failure results

**Figure 13:**
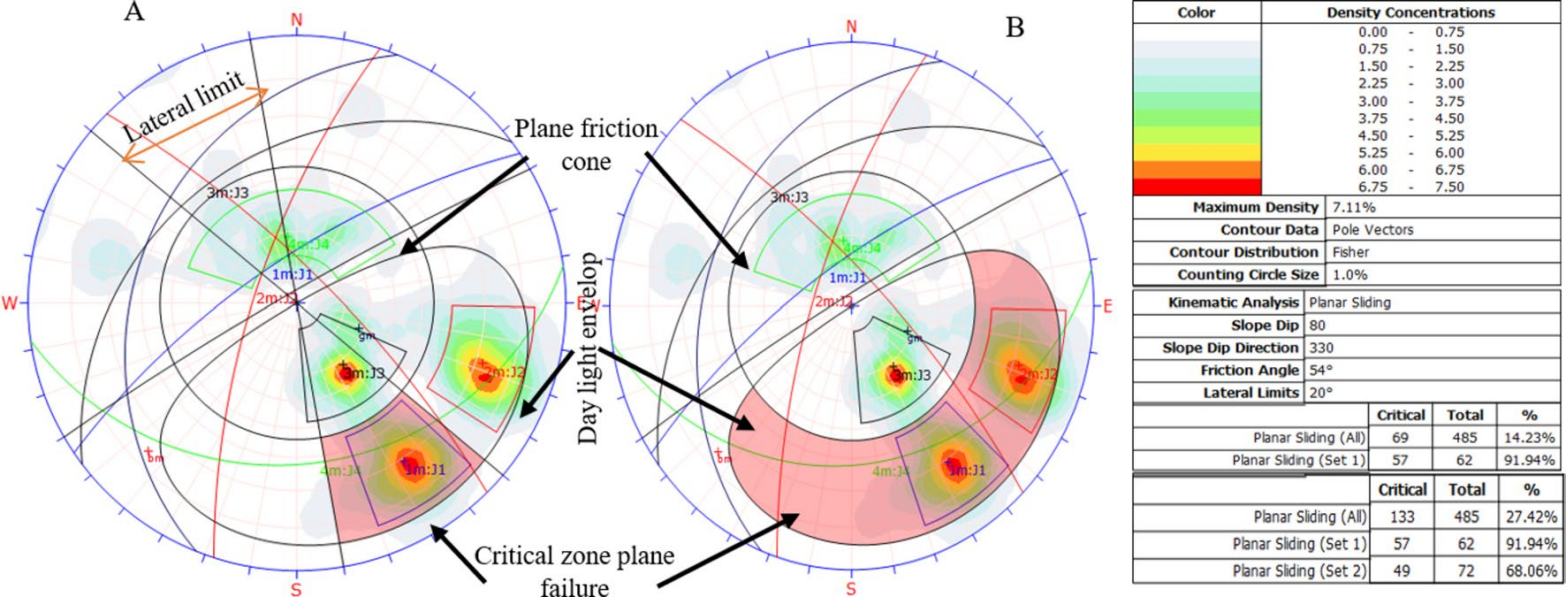
Planar failure with a 20% lateral limit (**A**) and with a free lateral limit (**B**).

Planar failure mode was predominantly influenced by the pole vector joint set and two major window joint sets. This is illustrated in Fig. 13A, where the critical zone is highlighted in red. The pole vector joint set had a density of 7.11%, with 14.23% (69 out of 485 poles) falling within the critical zone for planar failure. Figure 13B demonstrates that 91.94% of the J1 window joint set and 68.06% of the J2 joint set contributed to planar failure modes, as determined by kinematic analysis without lateral constraints. Additionally, the critical zone expanded to 27.24% for the pole vector joint set.

#### Toppling failure results

**Figure 14:**
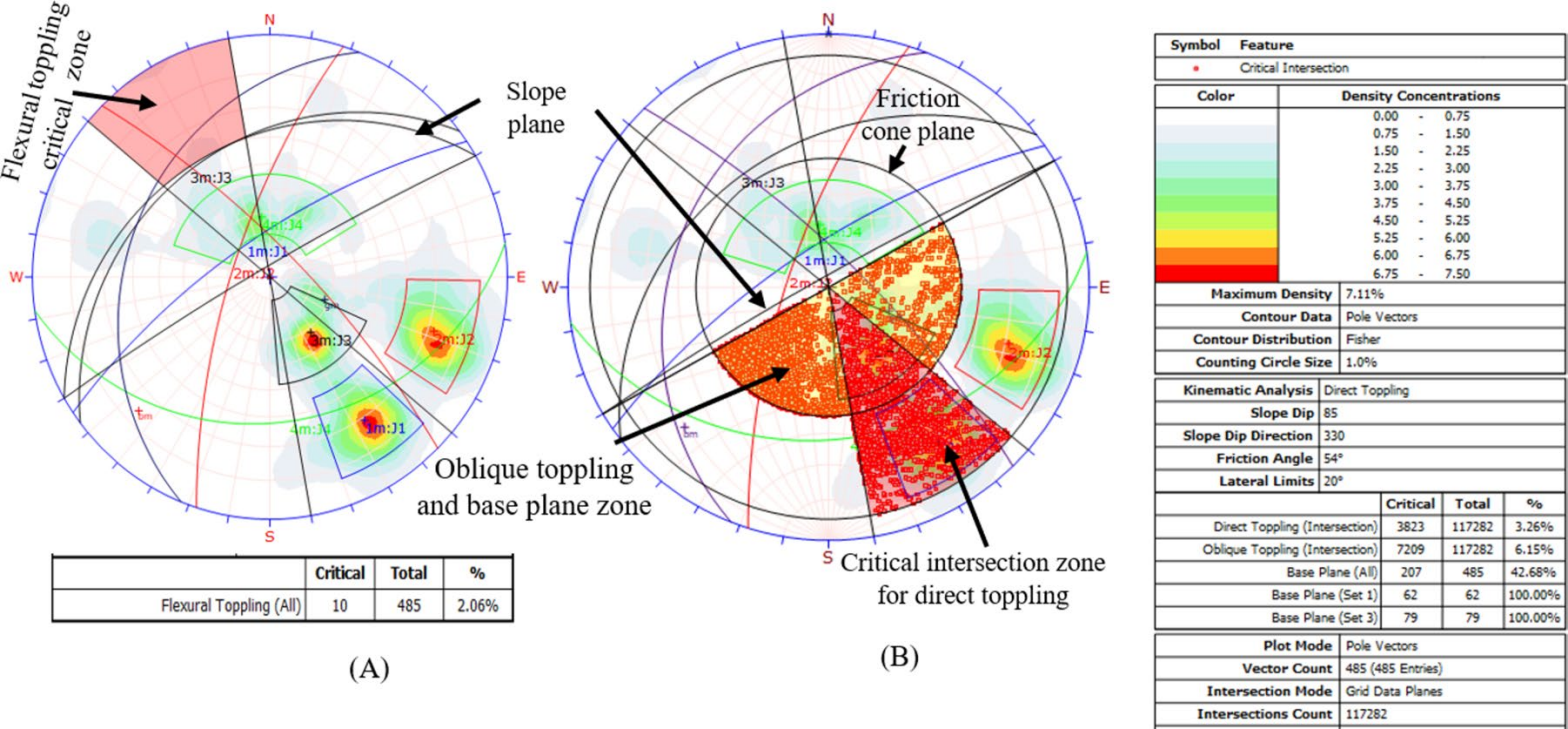
Modes of flexural toppling failures (**A**) and direct toppling failures (**B**).

The kinematic analysis conducted with the software identified two types of toppling failures flexural and direct. These failures happen when a slope plane dips below its friction angle, has material in the discontinuity, and leans outwards from the slope. Out of 485 examined discontinuity sets, only 10 were at risk of flexural toppling. Figure 14A, marked in red, shows that 2.06% of the samples could fail due to flexural toppling. Figure 14B indicates that 6.15% of intersections are nearly vertical and beyond the side limit, risking oblique toppling, while 3.62% of intersections dip within the slope and are at risk of direct toppling. Also, 42.68% of base plane intersections may fail by toppling, as depicted in Fig. 14.

### Discussion on modes of failure

The kinematic analysis showed that the studied quarry slope is unstable in several ways. Table 9 lists the types of failures caused by the interaction between the grid plane, major joint set openings, and various discontinuity planes.

**Table 9:** Summary of the failure modes and percent critical zone.

Failure mode	Intersection type	Critical (1)	Percentage (%)	Critical (2)	Percentage (%)	Total intersection
Wedge failure	Grid/base plane intersections	5207	4.44	17,696	15.09	117,282
	All set planes	1979	5.68	6234	17.88	34,859
	Joint set J1 versus joint set J2	1979	44.33	2383	53.38	4464
	Joint set J1 versus joint set J3	–	–	1595	32.56	4898
	Joint set J1 versus joint set J4	–	–	2256	39.13	5766
Toppling failure	All base plane vectors	207	42.68	–	–	485
	Joint set J1 versus Joint set J2			22	0.49	4464
	Joint Set J1 versus Joint set J4	–	–	18	0.31	5766
	Joint set J2 versus Joint set J4	–	–	895	13.37	6696
Plane failure	All vector (pole)	69	14.23	–	–	485
	Joint set J1	57	91.94	–	–	62
Free lateral limit)	All vectors (pole)	133	22.42	–	–	485
	Joint set J1	62	91.94	–	–	62
	Joint set J2	49	68.04			72

Critical 1 = Wedge Sliding (Both Planes) = Direct Toppling (Intersection).

Critical 2 = Wedge Sliding (One Planes) = Oblique Toppling (Intersection).

### Results of finite element analysis

This section outlines the numerical modeling used to assess the stability of current and projected quarry cut slopes, targeting the deepest limestone layers. It details the finite element analysis results, examining slope stability under various conditions, and compares these findings with baseline stability criteria. Additionally, it includes a parametric study of the quarry cut slope geometry, focusing on maximizing safety and resource efficiency.

#### Deformation analysis

**Figure 15:**
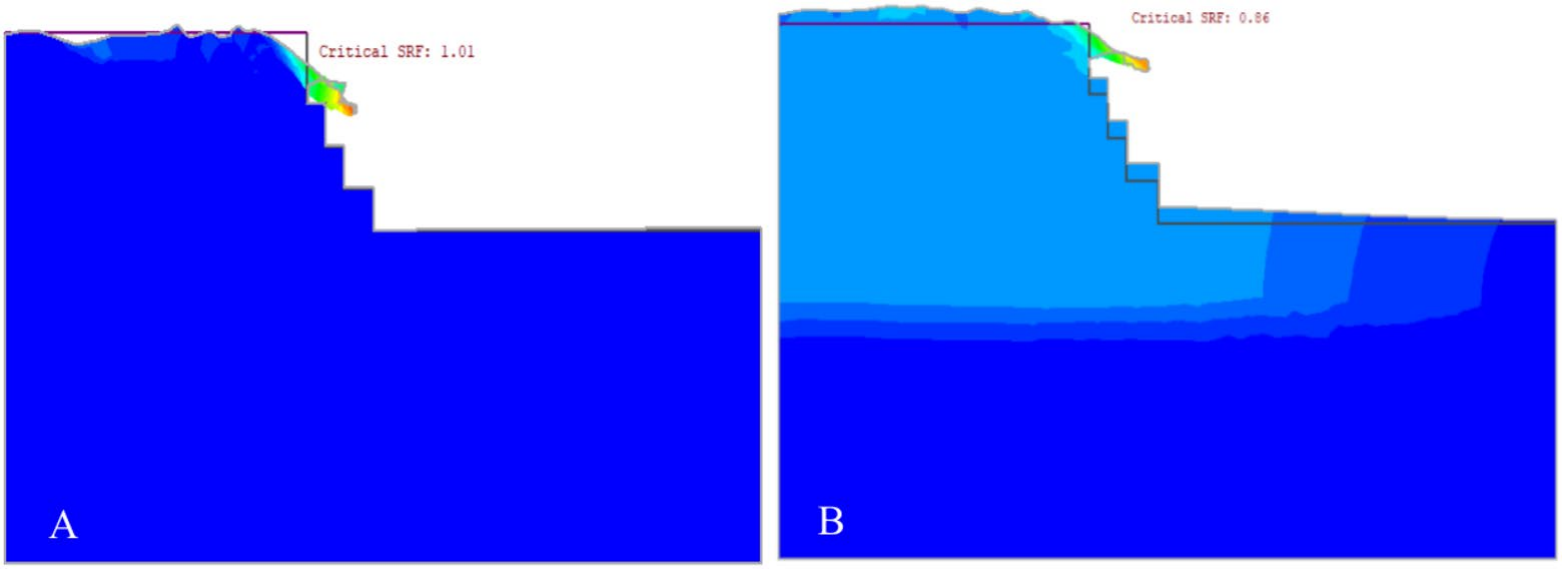
Deformation of existing quarry cut slopes due to static (**A**) and dynamic loading (**B**).

**Figure 16:**
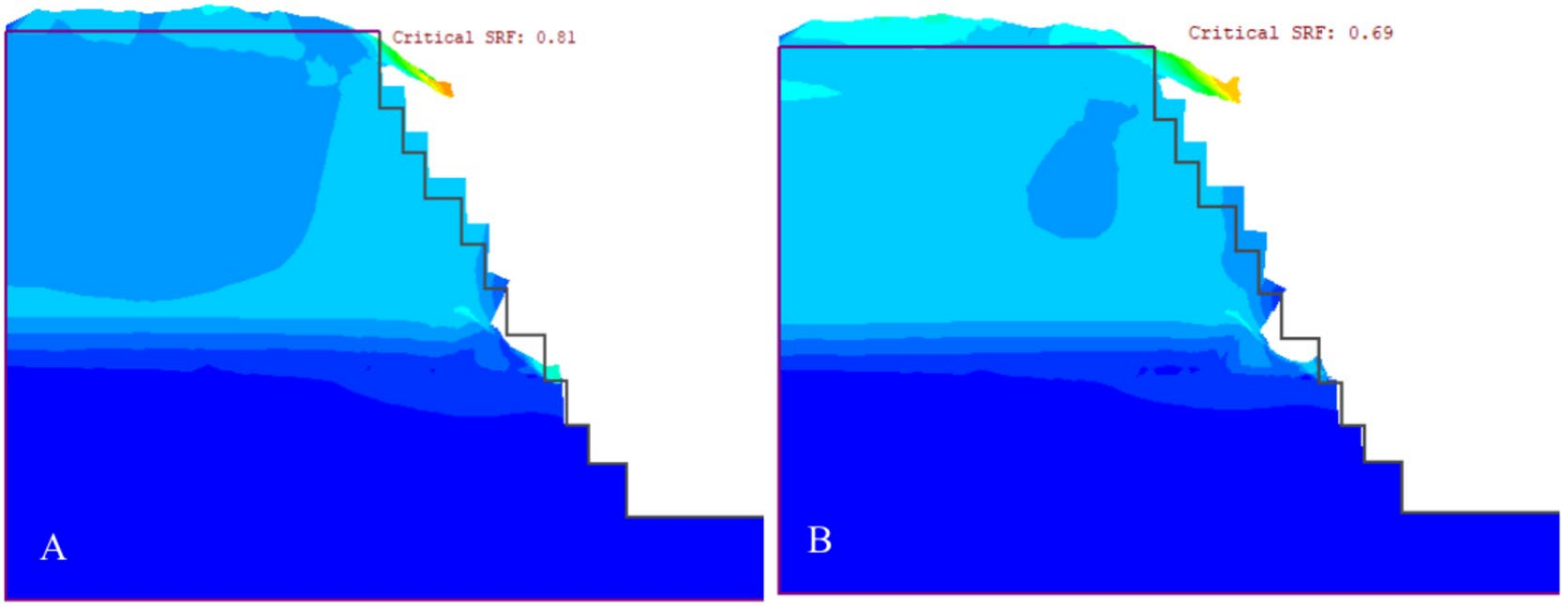
Deformation of the final quarry cut slope due to static (**A**) and dynamic loading (**B**).

The analysis of deformation in quarry slopes was conducted using plastic calculations. For static conditions, the load on each element was determined by its weight and the force of gravity, which also defined the stress in the field. During seismic events, deformation was assessed using a seismic coefficient derived from the ratio of maximum earthquake acceleration to gravitational acceleration. This coefficient introduced an extra force to the mesh elements, combined with gravity to form the total force on each element. For dynamic conditions, horizontal earthquake forces were applied as an acceleration coefficient (α = 0.115) for earthquakes and (α = 0.299) for blasts), while vertical acceleration was ignored due to its minimal impact (Figs. 15 and 16).

#### Deformation analysis results of the existing quarry cut slopes

**Figure 17:**
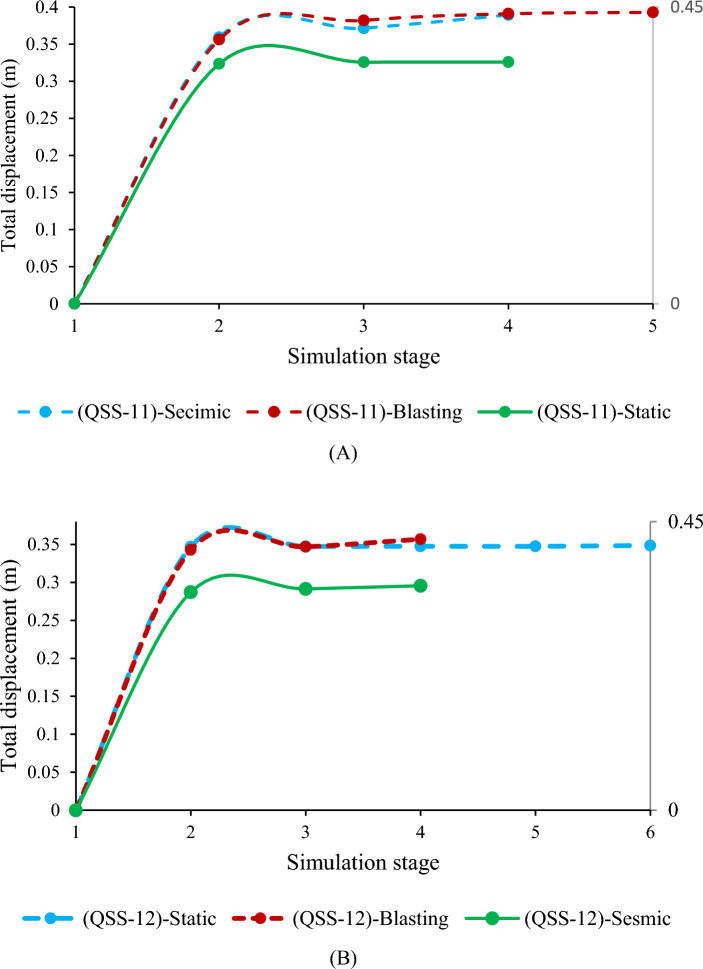
The relationship between the simulation stage and total displacement (**A** and **B**).

The study analyzed deformation in quarry cut slopes QSS-11 and QSS-12, subject to static and dynamic forces. Results from Phase2 finite element analysis revealed that under static conditions, QSS-11 and QSS-12 experienced total displacements of 36.65 cm and 34.83 cm, respectively (Fig. 17a,b).

The study evaluated how earthquakes and blasting vibrations affect slope stability. Using pseudo-static analysis, it was found that seismic activity with a horizontal acceleration of α = 0.115 g and blasting vibrations with α = 0.299 g increased the deformation of slope QSS-11 by 5.85% and 6.69%, respectively. Slope QSS-12 showed maximum displacements of 35.99 cm for seismic and 36.68 cm for blasting loads (Fig. 17). The study confirmed that both seismic and blasting vibrations contribute to increased slope deformation.

**Figure 18:**
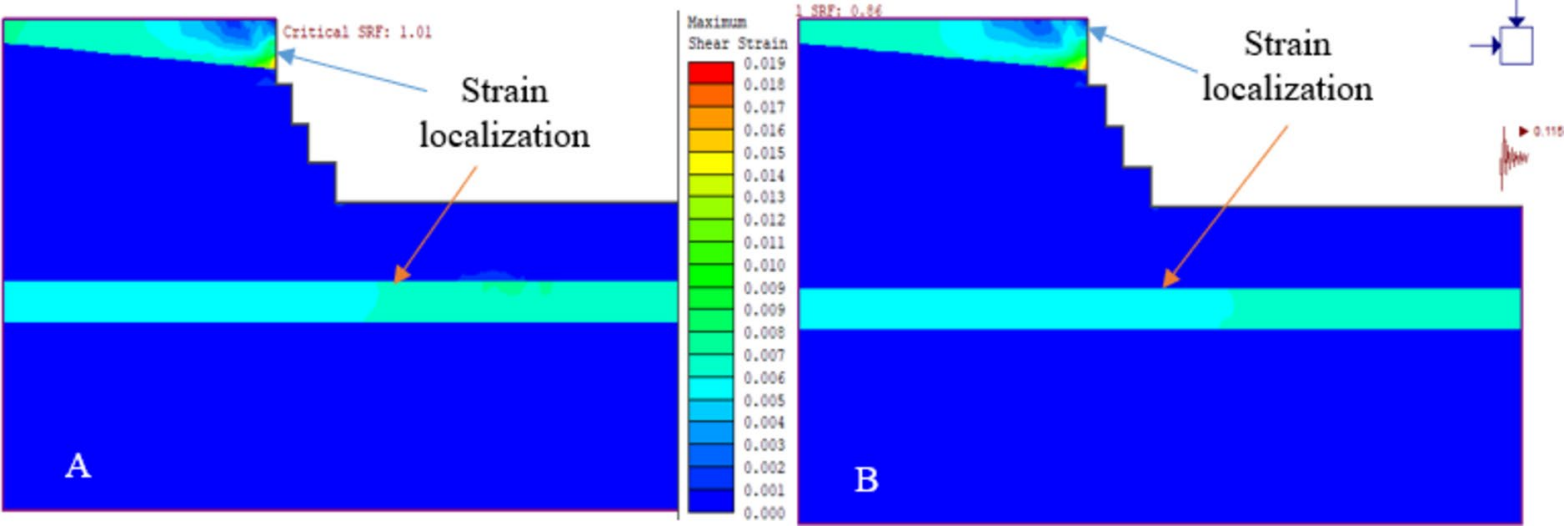
Maximum shear strain and SRF results for the existing quarry under static loading conditions (**A** and **B**).

Figure 18 indicates that the top layer of the slope, with weaker rock strength, deformed more significantly. This suggests a higher risk of failure in the slope layers, particularly due to weathering. These findings are consistent with field observations of slope failure.

#### Safety analysis results of the existing quarry cut slopes

**Figure 19:**
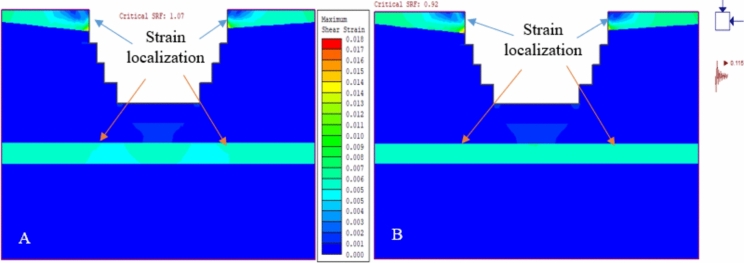
Maximum shear strain and existing SRF quarry for (**A**) static and (**B**) dynamic loading.

The study indicated that the SRF was 1.01 and 1.07 for the slope model under static conditions without additional load. When introducing pseudo-static analysis with seismic and blast vibrations, the SRF decreased^26^. Specifically, for the QSS-11 slope section, the horizontal seismic acceleration coefficient (α = 0.115 g) and peak ground acceleration (PGA = 0.29 g) saw reductions of 14.85% and 44.55%, respectively. The QSS-12 section exhibited similar trends, with decreases of 14% and 42%. The results, including the SRF for various loading scenarios, are detailed in Fig. 19 and Table 10.

**Table 10:** SRF and total displacement from Phase 2 software for QSS-11 and QSS-12.

Existing	Shear reduction factor (SRF)			Total displacement (cm)		
	Static	Pseudo static		Static	Pseudo static	
		Seismic (α = 0.115 g)	Blasting (PGA = 0.299 g)		Seismic (α = 0.115 g)	Blasting (PGA = 0.299 g)
Quarry Slope Section (QSS-11)	1.01	0.86	0.56	36.63	38.93	39.28
Quarry slope section (QSS-12)	1.07	0.92	0.62	34.86	35.99	36.68

#### Results for the ultimate depth of the existing quarry cut slopes

**Figure 20:**
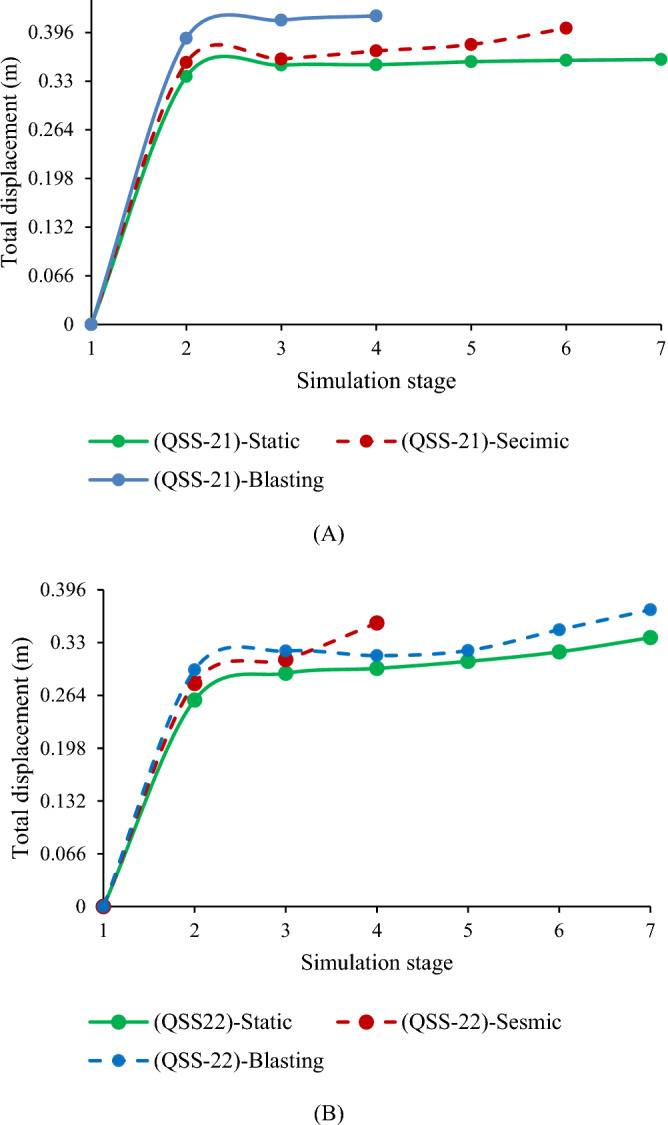
The relationship between the simulation stage and total displacement (**A** and **B**).

The study evaluated the effects of seismic activity and blasting on quarry slopes. Using pseudo-static analysis, it was found that earthquake vibrations (with a horizontal acceleration of α = 0.115 g and blasting vibrations α = 0.299 g increased the deformation of QSS-21 by 5.57% and 9.34%, respectively (Fig. 20a). QSS-22 experienced increases of 4.83% and 6.48% under the same conditions (Fig. 20b).

**Figure 21:**
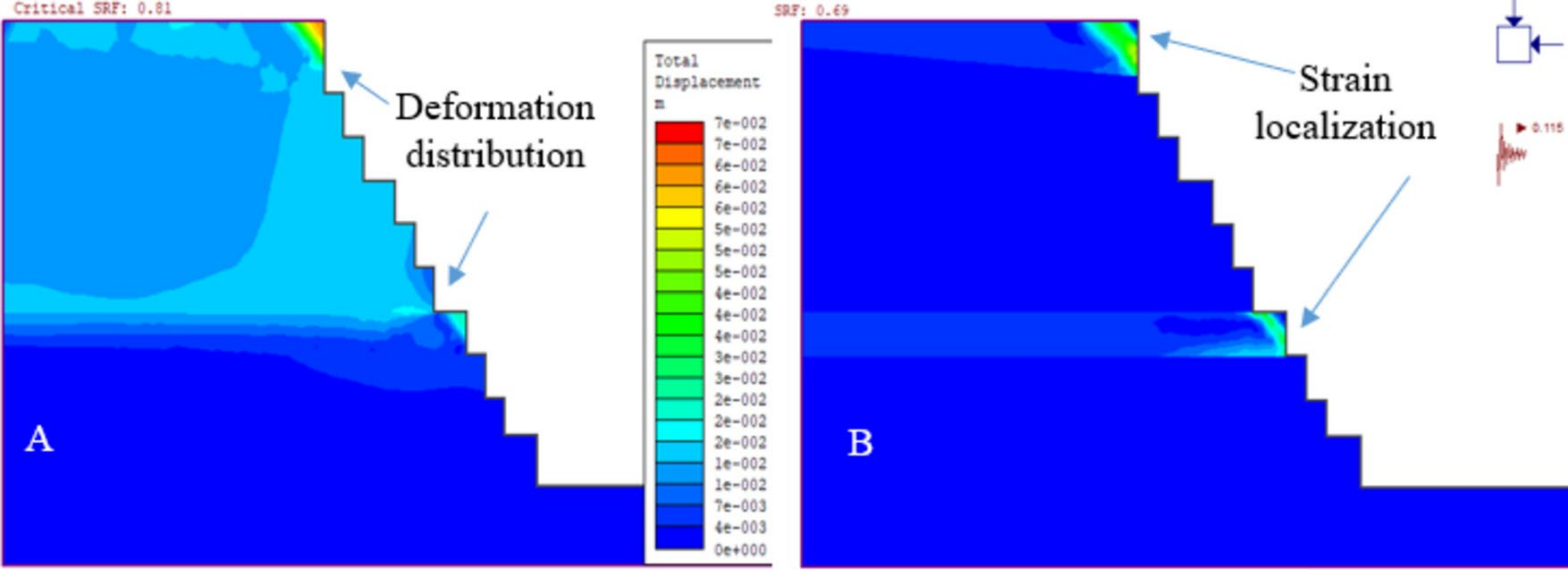
Deformation and SRF results for static (**A**) and dynamic loading conditions (**B**).

In this study, we analyzed the deformation of quarry slope sections QSS-21 and QSS-22, observing that an increase in slope height correlates with more pronounced deformation. This effect was especially noticeable in the weathered limestone of layers 1 and 5. Static condition assessments showed that QSS-21 and QSS-22 underwent significant displacement, with measurements of 37.94 cm and 35.64 cm, respectively, and QSS-22 exhibited marginally less deformation than QSS-21 (Figs. 20 and 21) respectively. The identified failure zone was predominantly located in the upper and middle layers of the weathered limestone, as depicted in Fig. 21.

#### Safety analysis results for the ultimate depth of the quarry cut slope

**Figure 22:**
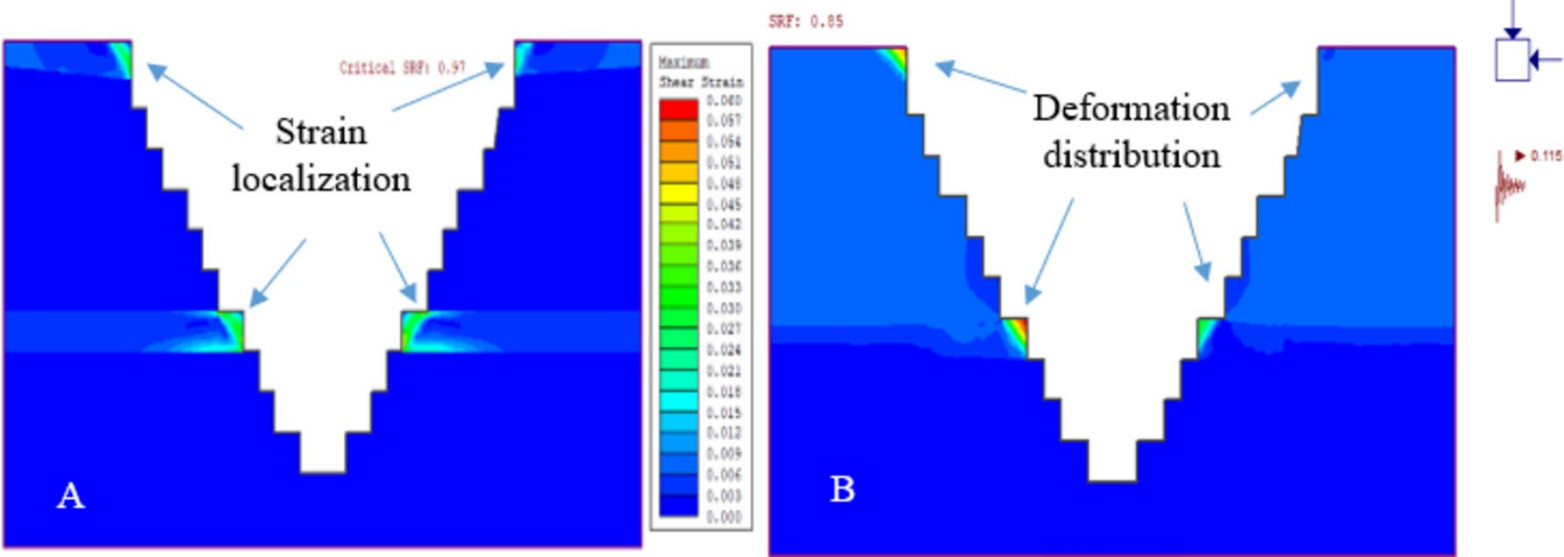
Maximum shear strain and SRF results for the static (**A**) and dynamic loading conditions (**B**).

The quarry slope stability was assessed for both static and dynamic conditions. The highest shear reduction factor (SRF) recorded was 0.97 for QSS-22 under static conditions. This factor reduced by 12.4% with seismic loading (α = 0.115) and 37.2% with blast vibrations (PGA = 0.299). For QSS-21, the highest SRF was 0.81, which decreased by 13.6% and 37% under seismic and blasting conditions, respectively, as illustrated in Fig. 22. The SRF analysis indicated that the slope is critically stable, primarily due to the weathered limestone in layers 1 and 5. For detailed values, refer to Fig. 22 and Table 11.

**Table 11:** Summary of the SRF and total displacement for the section of the ultimate limit slope.

Final quarry slope	Shear reduction factor (SRF)			Total displacement (cm)		
	Static	Pseudo-static		Static	Pseudo-static	
		Seismic (α = 0.115 g)	Blasting (PGA = 0.299 g)		Seismic (α = 0.115 g)	Blasting (PGA = 0.299 g)
Quarry slope section (QSS-21)	0.81	0.7	0.51	37.94	40.18	41.85
Quarry slope section (QSS-22)	0.97	0.85	0.61	35.64	37.45	38.11

#### Deformation vectors and plastic region distribution

**Figure 23:**
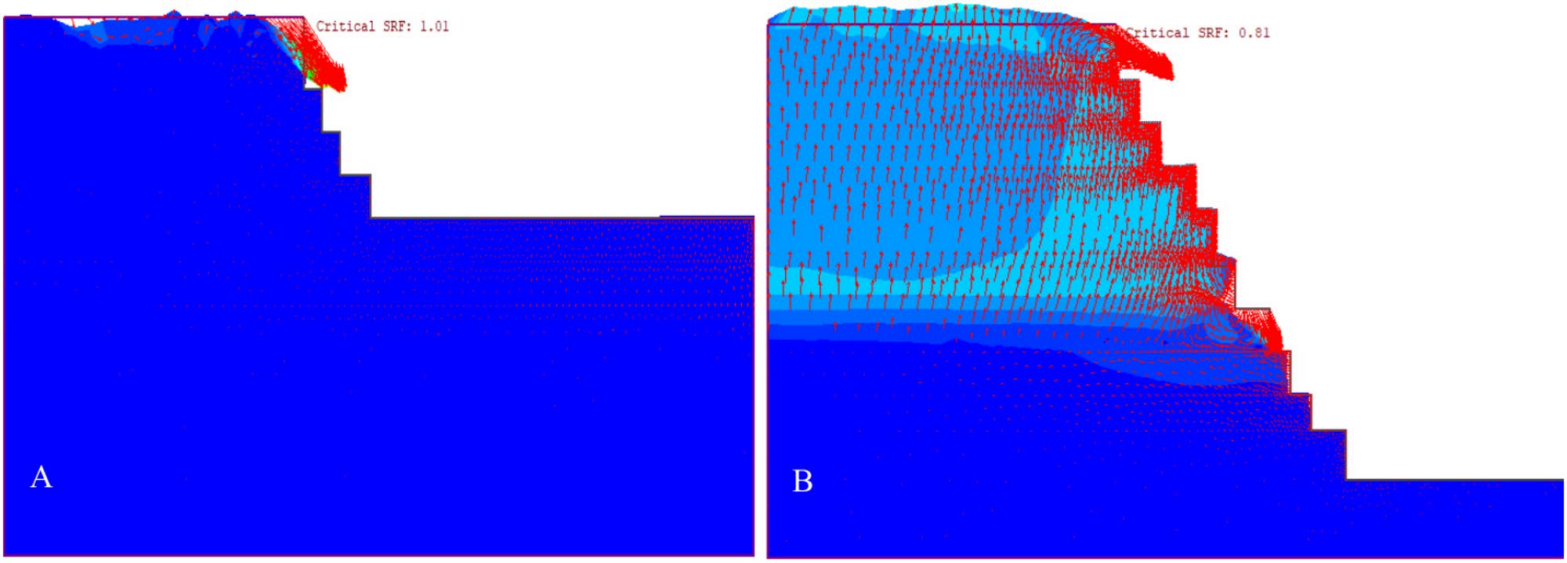
Distribution of the displacement vectors of the existing quarry (**A**) and the final slope of the quarry (B).

The study deformation vectors, illustrated in Fig. 23, show the movement and potential collapse patterns of the slopes. These vectors indicate a noncircular failure mode common in rock slopes, where the material shear strength dictates failure over any discontinuities. Thus, the slope movement during failure is consistent across different models.

**Figure 24:**
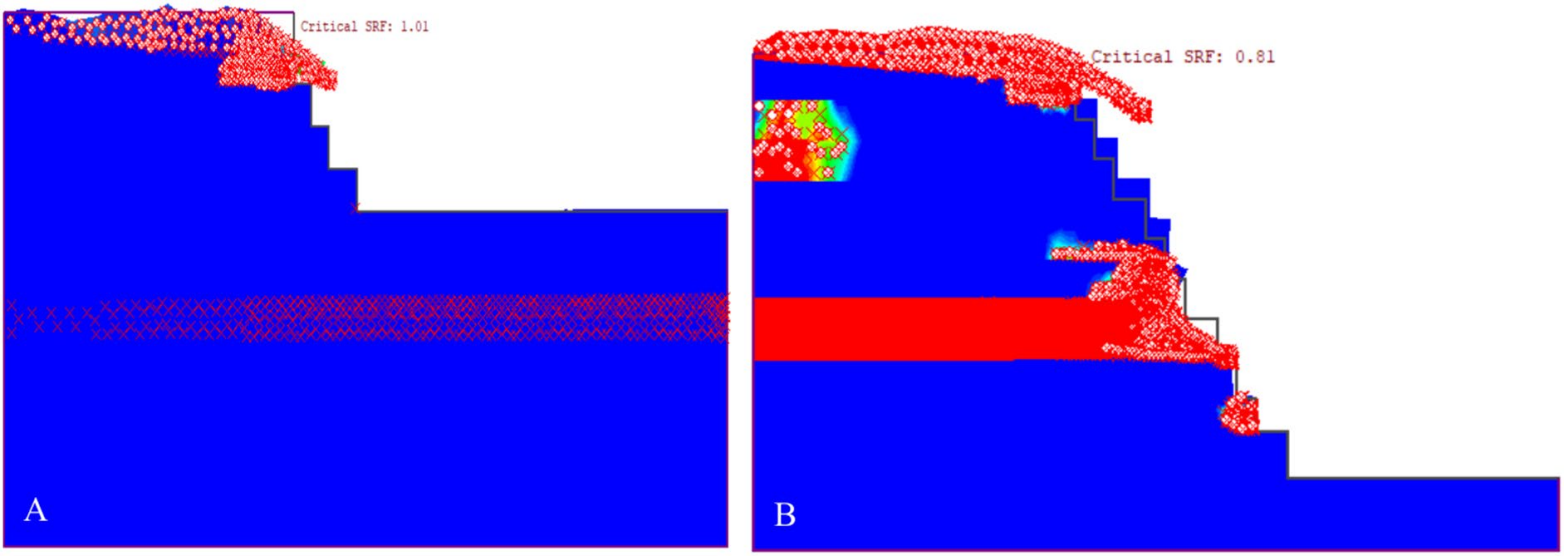
Propagation of the plastic region of the existing quarry slope (**A**) and the final quarry slope (**B**).

Figure 24 deformation vectors depict the slope failure modes, with white circles indicating tension failures and red stars denoting shear failures. The distribution of these failures deviates from the areas of maximum shear stress. Notably, the failures are prevalent within the highly weathered limestone of the slope middle and upper layers, highlighting this as the primary failure mechanism.

#### Discussion of finite element analysis

The numerical analysis was conducted to assess the stability of the quarry slopes, which is essential for selecting a stable and cost-effective design. The analysis indicated a potential failure surface and maximum deformation, with a minimal SRF for the current slope. The SRF was low 0.06 for both models, suggesting a negligible impact on design. However, the discrepancy increased under pseudo-static analysis.

**Figure 25:**
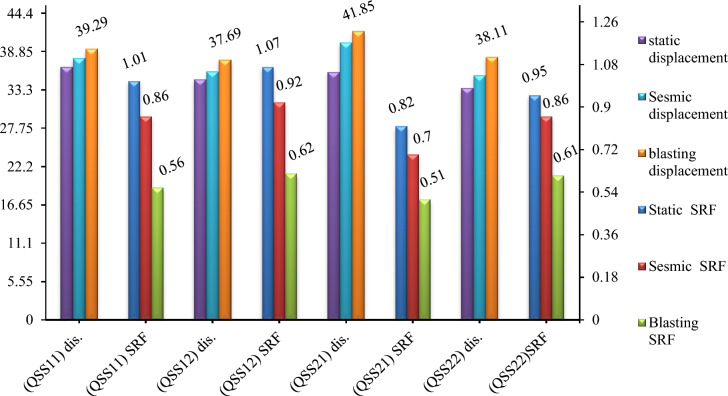
Summary of the total displacement and SRF of the selected slope profile under the different loading conditions.

Furthermore, the deformation and stability of slope sections QSS-21 and QSS-22 were comparable to those of QSS-11 and QSS-12, with SRF of 0.81 and 0.97. The variations were marginally higher in the recent analyses. In general, the quarry cut slopes exhibited lower SRF and deformation than the open-pit models. Although this warrants further discussion, the focus of this study was on quarry cut slopes for the parametric analysis, aiding in the design of optimal excavation depths. The deformation factor and safety outcomes from the numerical analysis are depicted in Fig. 25.

#### The stability condition of the slope

**Figure 26:**
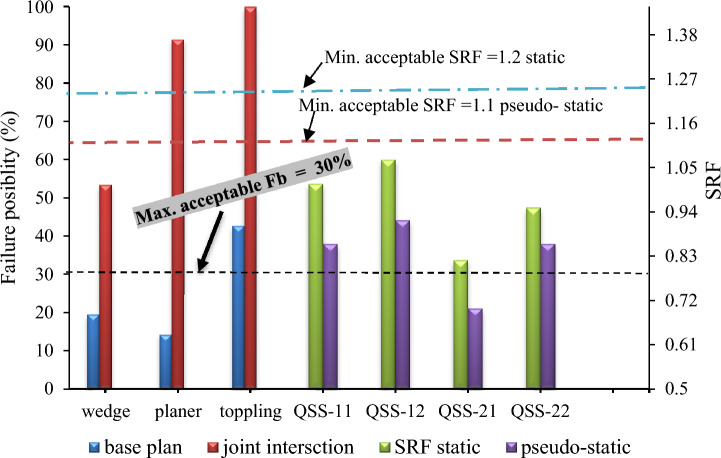
Summary of the failure possibilities and SRF for static and seismic loading.

In assessing the safety and functional design of excavated slopes such as open pit mining and road cuts and natural slopes, rock slope stability analyses are crucial. This study employed two basic stability analysis methods to explore potential failure mechanisms and slope stability conditions. Notably, if kinematic analysis indicates more than 30% counter generation for any failure mode, it is considered critical^50^. The kinematic analysis results revealed that the quarry slope section was susceptible to wedge, planar, and toppling failure modes, as depicted in Fig. 26.

The study simplified the concept of slope safety, stating that slopes are generally safe if the factor of safety exceeds 1. This is due to uncertainties in material analysis, boundary conditions, and computational methods. References^15,50,51^ suggest a minimum SRF of 1.2 to 1.3 for static conditions and 1.1 for dynamic conditions. The quarry critical slope had an SRF between 1.01 and 1.07, making it marginally stable but below the recommended SRF. Under seismic conditions, the SRF fell to 0.86 and 0.56, indicating instability.

The study found that the final quarry slope SRF was below 1, indicating instability and a high risk of failure. The SRF values were also below the minimum standard (SRF > 1.2) for static conditions. Figure 26 shows that the potential for failure, including wedge, planar, and toppling, exceeded safe limits, and FEM analysis confirmed these findings for both static and pseudo-static conditions, pointing to a critical state of failure for the slope.

### Parametric study of the quarry cut slope profiles

Slope geometry is pivotal for stability, assessed through safety factors and deformation. Segmenting the slope into smaller parts improves stability by optimizing the SRF and preventing shallow slopes. Stability varies with bench dimensions and face angle. The study analyzed multi-bench quarry slopes by altering individual parameters like face angle, bench size, and inter-ramp angle. Using Phase2 FEM analysis determined deformation and SRF, focusing on parameter impacts on stability, safety, operational access, and cost-efficiency.

#### Effects of the bench width on the maximum deformation

**Figure 27:**
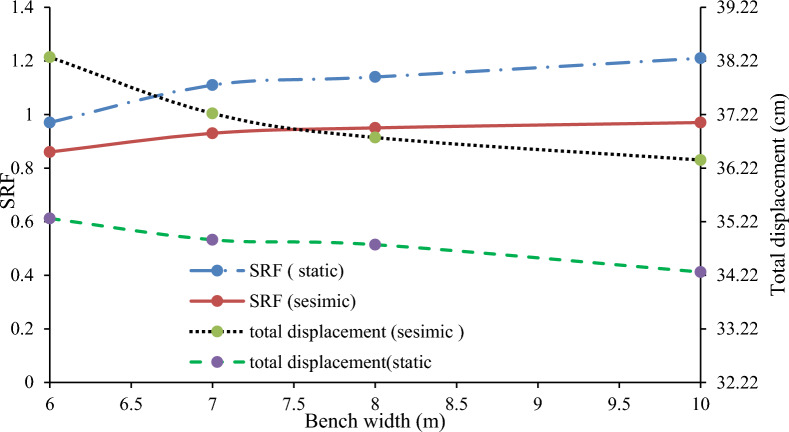
Relationship between the bench width on the SRF and the deformation.

The stability of the quarry cut slope improves with wider benches. Modeling revealed that increasing bench width from 6 to 7 m, 8 m, and 10 m enhances Factor of Safety (FS) by 12.6%, 14.9%, and 19.8%, respectively, and reduces deformation (Fig. 27). This is due to the reduced driving force above the failure surface with wider benches.

#### Effect of the bench height on the maximum displacement

**Figure 28:**
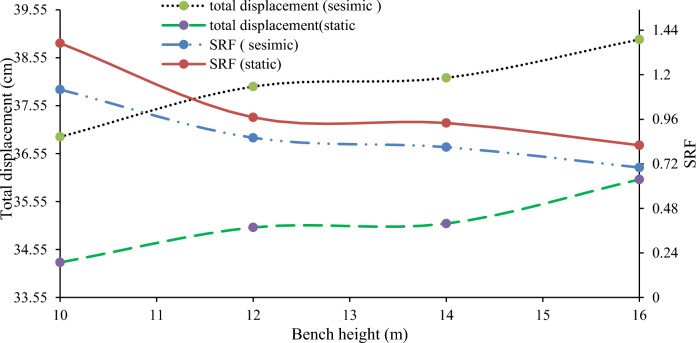
Relationships between the height of the cut-slope bench, the maximum displacement, and the SRF.

The stability of a slope is influenced by its angle and height, which affect shear stress and strength. Large open-pit mines typically use bench heights ranging from 10 to 18 m. An analysis of slopes with a fixed bench face angle of 90° and varying heights (10, 12, 14, and 16 m) showed that higher benches result in lower SRF and increased displacement due to greater vertical stresses (Fig. 28).

#### Effect of the slope face angle on the deformation

**Figure 29:**
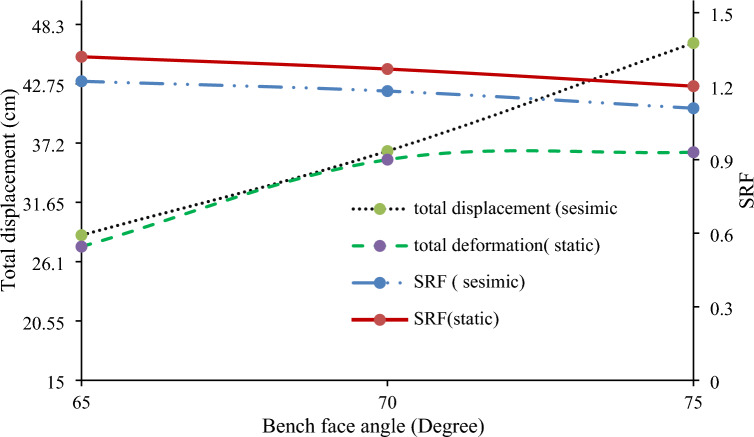
Relationships between the cut-slope face angle, maximum displacement, and SRF.

Slope stability is significantly affected by the slope face angle. Numerical models with a 6 m bench width and 12 m bench height revealed that angles of 65°, 70°, and 75° decrease the SRF and increase displacement (Fig. 29). Reducing the slope face angle to below 70° enhances stability and meets the safety factor requirement of 1.2, suggesting a flatter slope for ongoing construction.

#### Effect of inter-ramp angle on deformation

**Figure 30:**
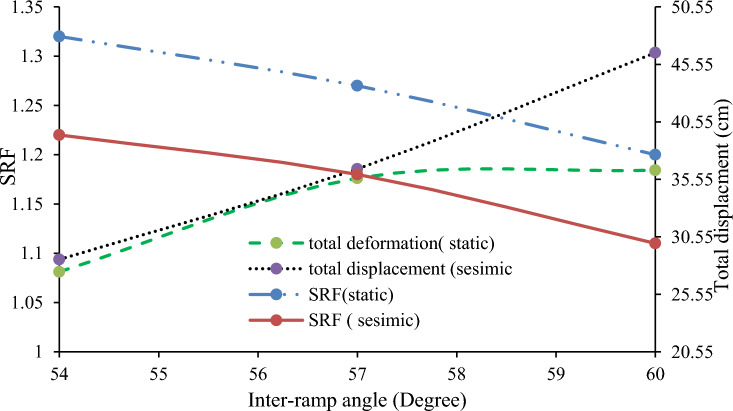
Relationship between the inter-ramp angle to the SRF and the deformation at a constant bench height.

**Figure 31:**
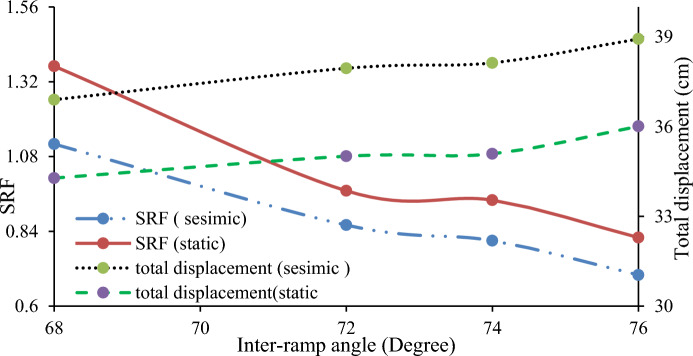
The relationship between the angle of the inter-ramp and the deformation is a constant face height.

Inter-ramp angles, formed by benches with varying slopes, impact the stability of both the intermediate structure and the overall slope. A study Hustrulid^52^ indicated that structures influencing inter-ramp stability must have a cutoff length at least as long as the height of two design benches. Numerical models tested two scenarios to assess these effects. In Case 1, a quarry slope with varying bench heights and fixed bench face angles (68°, 72°, 74°, and 76°) showed that steeper inter-ramp angles led to lower SRF and higher displacements (Fig. 30). Case 2, with uniform bench height and varying bench face angles (54°, 57°, and 60°), confirmed that steeper angles adversely affect SRF and displacement (Fig. 31).

### Selection of design options

**Figure 32:**
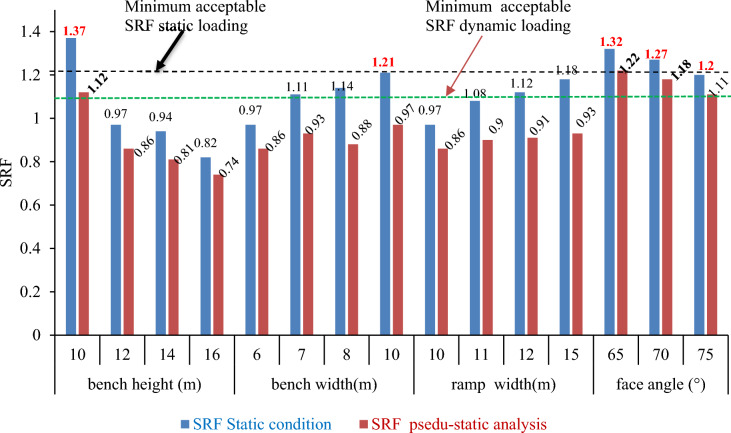
Summary of SRF for parametric study in static and pseudo-static analysis.

The primary goal of effective quarry design is to minimize the risk of failure and ensure safety during operations. Simultaneously, quarry operators aim to maximize mineral reserve extraction. Achieving this involves designing steep quarry faces, narrow benches, and high cut faces while mitigating instability risks. In section “Results of finite element analysis”, we compare parameters that influence quarry slope geometry, resource extraction, and stability. Figure 32 illustrates the SRF results, highlighting the significant impact of modifying these geometric parameters on overall safety, operational efficiency, and resource management.

The empirical study revealed that only three quarry slope profiles with a bench height of 10 m, bench width of 10 m, and cut face angles of 65°, 70°, and 75° satisfied the minimum stability criteria (SRF > 1.2) under static conditions. Additionally, four profiles with bench heights of 10 m and cut face angles of 65°, 70°, and 75° met the stability criteria (SRF > 1.1) under pseudo-static conditions, accounting for seismic effects. The goal was to select the safest design for quarry operations, prioritizing the steepest possible cut slope face angle and the narrowest benches to maximize reserve extraction. The analyses for quarry slope safety and resource management were then compared.

1. Figure 32 shows how the multi-bench quarry slope is affected by the reduction in the face angle. The reserves lost per 1 m of cut face width increase from a bench height of 12 m to 33.64 m^3^ as the face angle decreases from 90° to 65°.
2. Figure 32 shows how the quarry cut slope with a face angle of 70° is affected by the reduction in the bench face. The reserves lost per 1 m width excavation increase from 12 m in height to 26.27 m^3^ as the bench face decreases.
3. Figure 32 shows how the reduction of the bench face affects the quarry cut slope with a face angle of 75°. The reserves lost per 1 m of quarry cut width increase from 12 m in height to 19.25 m^3^ as the bench face decreases. This leads to a minor loss of resources but compromises human life and property.
4. Excavation of hard rock masses with lower bench heights was not profitable because it decreased the production yield tonnage during the blast explosion. Therefore, as shown in Fig. 32, lowering the bench height of the quarry cut slope reduced the limestone production by 96.25 tons per hole.

**Figure 33:**
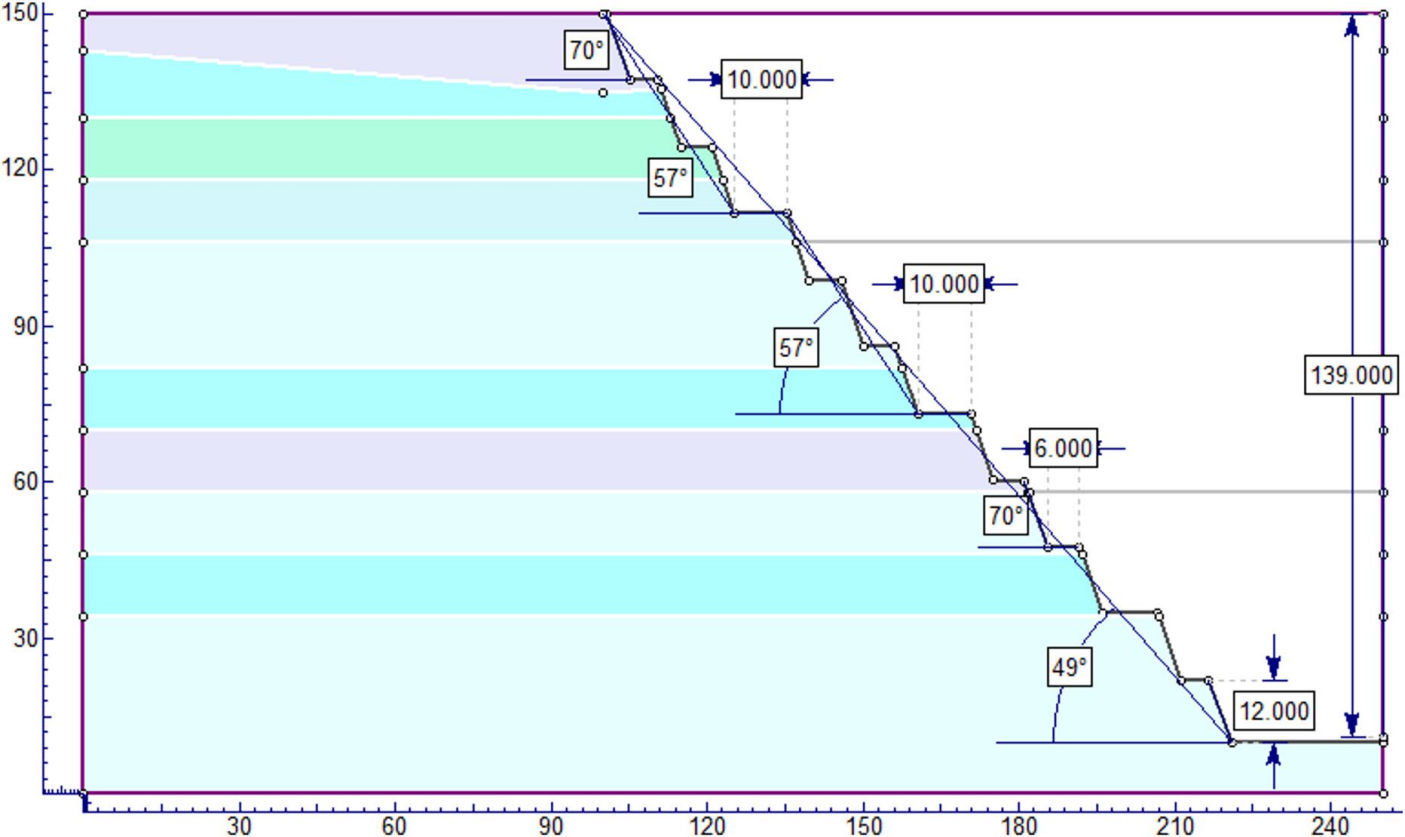
Summary of geometric profiles for selected optional quarry cut slopes.

The calculation was based on short quarry slopes (12 m) and simple geometry. To reflect the quarry true size, the figures would be significantly larger, affecting its viability. The best design balanced safety and reserve loss, as shown in Fig. 33.

## Conclusions

This study focuses on the ongoing quarry excavation at the National Cement Factory in Dire Dawa city, which has reached a depth of over 56 m with significant signs of slope failures. The quarrying aims to reach a depth of 150 m. The study evaluates the stability of the current and projected quarry slopes and the impact of geometric profiles on slope stability. Geotechnical parameters were identified through field investigations, tests, and empirical correlations using field data. The steep dip of the slope and discontinuities suggest a high probability of instability. Kinematic analysis using DIPS software identified potential failure modes, showing varying degrees of instability. Stability evaluations for all quarry slope configurations were conducted under static and dynamic conditions using finite element analysis Phase2. The conclusions from these analyses are as follows:

- Kinematic analysis revealed a high risk of toppling failure for the quarry cut slope, with a probability of 42.68%. The risks of wedge and plane sliding failures were lower, at 19.53% and 14.23%, respectively, at the base plane intersection.
- Finite element analysis showed that the SRF for the current quarry slope section was 1.01 under static conditions. However, it decreased to 0.86 under seismic effects and 0.56 under blasting vibrations. These results indicate that the quarry slope is unstable under both static and dynamic loading conditions.
- The shear reduction factor for the quarry cut slope section at the projected final depth was 0.82 under static loading conditions. However, it decreased to 0.7 under seismic loading and further to 0.51 under blasting effects. These results indicate that the design of the quarry cut slope needs to be modified to improve stability.
- The study aimed to identify the optimal design by assessing various geometric profiles. It concluded that modifying geometry is a more effective and cost-efficient method for improving slope stability than other techniques. Additionally, a parametric analysis of quarry slope profiles was performed.
- Reducing the height and angles of the bench, along with increasing the width of the bench and ramp, enhances the stability of the quarry cut slope. This approach minimizes safety hazards but also limits the potential to extract the maximum amount of rock reserves.
- The stability of the quarry cut slope is significantly influenced by the angle of the bench face. When the face angle was reduced from 90° to 75°, 70°, and 65°, the shear reduction factor increased by 31.6%, 35.4%, and 37.9%, respectively, while maintaining the bench height at 12 m.
- Reducing the face angle of a multi-bench quarry slope increases the reserves lost per meter of cut face width. As the face angle decreases from 90° to 65°, the reserves lost rise from 12 to 33.64 m^3^. For face angles of 70° and 75°, the reserves lost increase to 26.27 m^3^ and 19.25 m^3^, respectively. This reduction in bench face height poses significant risks to human life and property and is not profitable, as it reduces production yield during blasting. Lowering the bench height decreases limestone production by 96.25 tons per hole.

## Data Availability

The data used to support the findings of this study are included within the article.
